# A chemically fueled non-enzymatic bistable network

**DOI:** 10.1038/s41467-019-12645-0

**Published:** 2019-10-11

**Authors:** Indrajit Maity, Nathaniel Wagner, Rakesh Mukherjee, Dharm Dev, Enrique Peacock-Lopez, Rivka Cohen-Luria, Gonen Ashkenasy

**Affiliations:** 1 Department of Chemistry, Ben-Gurion University of the Negev, 84105 Beer Sheva, Israel; 2 Department of Chemistry, Williams College, Williamstown, MA 02167 USA; 3 Present Address: Institute for Macromolecular Chemistry, Albert Ludwigs University of Freiburg, D-79104 Freiburg, Germany; 4 Present Address: Institute of Chemical Sciences and Engineering, Ecole Polytechnique Fédérale de Lausanne, 1015 Lausanne, Switzerland

**Keywords:** Origin of life, Dynamic combinatorial chemistry

## Abstract

One of the grand challenges in contemporary systems chemistry research is to mimic life-like functions using simple synthetic molecular networks. This is particularly true for systems that are out of chemical equilibrium and show complex dynamic behaviour, such as multi-stability, oscillations and chaos. We report here on thiodepsipeptide-based non-enzymatic networks propelled by reversible replication processes out of equilibrium, displaying bistability. Accordingly, we present quantitative analyses of the bistable behaviour, featuring a phase transition from the simple equilibration processes taking place in reversible dynamic chemistry into the bistable region. This behaviour is observed only when the system is continuously fueled by a reducing agent that keeps it far from equilibrium, and only when operating within a specifically defined parameter space. We propose that the development of biomimetic bistable systems will pave the way towards the study of more elaborate functions, such as information transfer and signalling.

## Abstract (web summary)

Although bistability is common in biology, it is very difficult to design de novo into synthetic systems. Here, the authors present an experimental and theoretical analysis of a chemical network that displays bistable behavior under certain far-from-equilibrium conditions, and map the parameter space in which bistability operates.

## Introduction

Biological systems, such as yeasts and bacteria, have long been used for manufacturing chemicals and materials. The macromolecular networks that control these miniaturised ‘factories’ can also be programmed—via the exploitation of synthetic biology tools—to perform complex functions foreign to the cell cycle, yielding, for example, oscillatory or chaotic behaviour^1,2^. Remarkable developments in Systems Chemistry over the past decade offer alternative strategies for the bottom-up design of entirely synthetic molecular networks with life-like functions^3–6^. While a systems chemistry approach may sometimes appear to be challenging with respect to design and synthesis, it often facilitates better control over a desired function and subtle manipulations, together with widening the range of experimental conditions. Significantly, recent progress in the systems chemistry research towards life-like behaviours has been achieved via the development of schemes for operating out of equilibrium, which have so far provided access to transient structures that form as a result of constant energy dissipation^7–16^. We now propose that the study of chemical networks out of equilibrium would further allow control over dynamical network-dependent functions. We thus report here thiodepsipeptide-based non-enzymatic networks propelled by reversible replication processes out of equilibrium, presenting bistable behaviour. Bistability is reached due to a disparity between forward and backward processes and is accessible only as long as energy is supplied by a reducing agent. Using theory, simulation and experiments, we characterise the bistable behaviour of the networks under a wide range of conditions and establish the limits and requirements for phase transitions from ‘ordinary’ behaviour—leading to a single steady state—to bistable functionality. We envision that the development of biomimetic bistable systems will pave the way to the study of more elaborate functions in synthetic networks, such as information transfer and signalling.

**Fig. 1:**
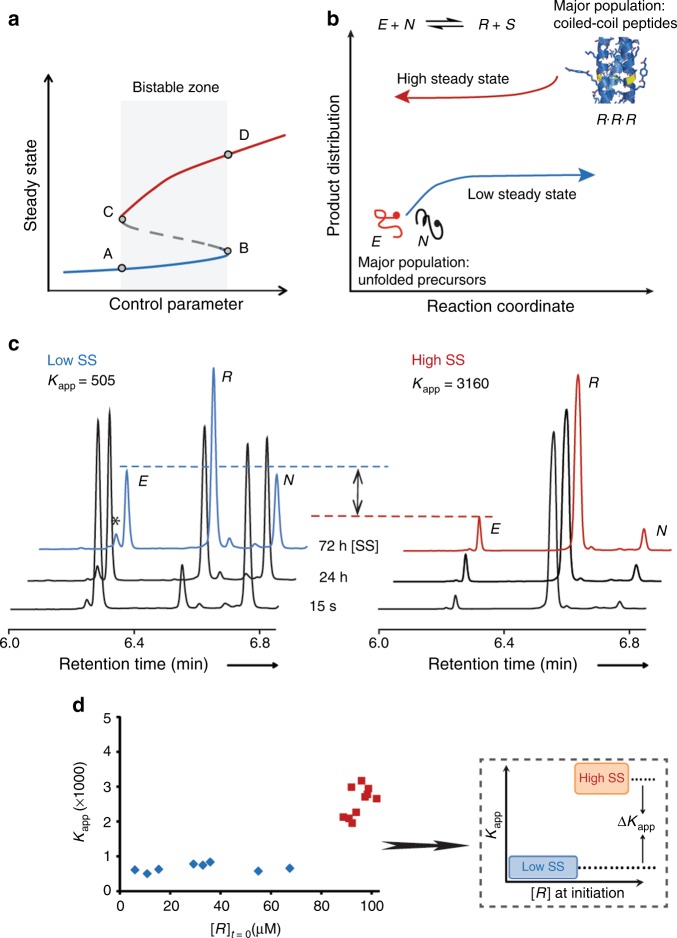
Bistability in a thiodepsipeptide replication network. **a** A typical bifurcation diagram describing the bistable response to changes in the level of a control parameter. The bistable zone is indicated by the shaded area; the blue and red lines signify the low and high SSs, respectively; A and D indicate the bifurcation points where phase transitions from two stable SSs to one SS, or vice versa, take place. **b** Scheme describing the network experiment that invokes bistability in our system. When the replicating reaction is initiated with high concentrations of unfolded precursors ***E*** and ***N***, the network reaches a low SS, whereas initiating the reaction with a high concentration of the folded replicator ***R*** leads to a high SS. The following peptide sequences were used: ***E*** = Ar-RVARLEKKVSALEKKVA-COSR, ***R*** = Ar-RVARLEKKVSALEKKVAZLEXEVA-RLKKLVGE-CONH_2_. ***N*** = H-ZLEXEVARLKKLVGE-CONH_2_. ***S*** = SR = 2-mercapto-ethane sulphonate, Ar = 4-acetamidobenzoate, Z = SCH_2_CO and X = Lys-Ar. **c** UPLC traces showing the reaction kinetics of experiments that were initiated either with 90 µM ***E***, 90 µM ***N*** and 10 µM ***R***, or with 5 µM ***E***, 5 µM ***N*** and 95 µM ***R***, leading to low and high SS product distributions, respectively. Experiments were carried out in the presence of 10 mM of a thiol molecule (2-mercapto-ethane sulfonate sodium salt) at room temperature (22 °C). **d** Typical bistable picture obtained by plotting *K*_app_ as a function of the initial [***R***], showing low (blue) and high (red) SS distributions. Experiments were carried out at a concentration of total peptides ([***E***] + [***R***]) of 100 µM under the same conditions as in **c**. The detailed SS concentration results are shown in the Supplementary Tables 1 and 2 (Entries #9). The insert shows a schematic representation of the difference between average *K*_app_ values expressed by ∆*K*_app_

**Fig. 2:**
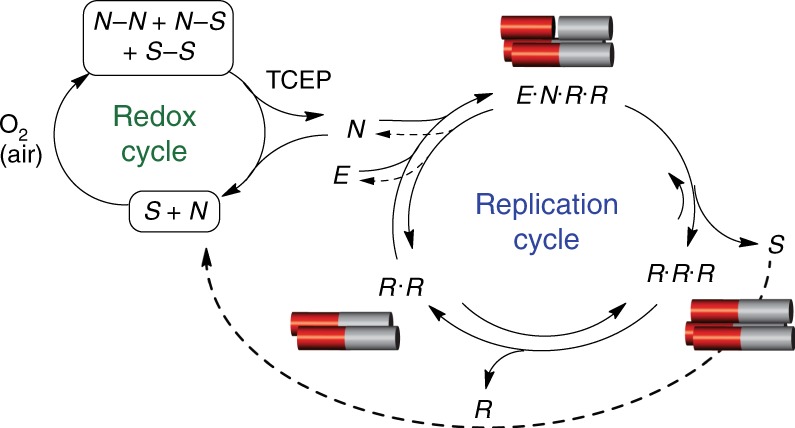
Coupled redox-replication cycles affording bistability. A chemically fuelled replication network, in which excess TCEP continuously drives disulphide reduction in the redox cycle, forming free thiol species that, in turn, facilitate reversible processes within the replication cycle

Bistability is often manifested by cellular networks, in which bistable motifs yield long-term memory, converting transient signals or stimuli into sustained responses that control different processes, including cell division, apoptosis and differentiation^17,18^. Sustained bistable responses can also form in self-organised chemical systems residing out of equilibrium, when two long-lived states, designated steady states (SSs), are alternately populated depending on the network rewiring in response to variations in a control parameter (Fig. 1)^19^. Despite recent progress in studying bistable (and multi-stable) systems by using small molecules^20,21^, gel materials^22^, and synthetic biology^23–25^, the de novo bottom-up design of such systems remains challenging, primarily because the kinetic characteristics and energy aspects yielding bistability have not been globally defined. After several research groups, including our own, have shown that synthetic replication networks can be wired and manipulated to perform simple functions observed in the biological context^26–30^, we describe herein an exceptional non-enzymatic network presenting bistability, driven by a reversible autocatalytic reaction of coiled-coil thiodepsipeptides^26^. We present here a quantitative analysis of the bistable behaviour^31–33^, featuring a phase transition from the simple equilibration processes taking place in reversible dynamic chemistry into the bistable behaviour observed only when the system is continuously fuelled (in this case by a reducing agent) to keep it far from equilibrium (see mechanism in Figs. 1b and 2) and when it operates under a specifically defined parameter space.

## Results

### Design principles of the bistable system

The second-order autocatalysis operating in this reaction constitutes the positive feedback^34^ that leads to the non-linear growth of a full-length thioester replicator (***R***)^35^, reversibly synthesised from its precursors, a thioester electrophile (***E***) and a thiol-containing nucleophile (***N***; Fig. 1b–d). The bistable behaviour is manifested in two distinct SSs of the obtained product distribution, either high or low, depending on the initial relative concentrations of ***R***, ***E***, and ***N*** (Fig. 1d). Mechanistically, we explain the bistable behaviour by the disparity between the fast interconversion of (unfolded) ***R*** to ***E*** and ***N*** molecules in a template-free reaction, versus the slow interconversion in a template-assisted reaction in which ***R*** is assembled into a well-folded trimeric coiled-coil nano-structure, which can react only slowly with incoming thiols (Fig. 1b)^26^. The observed SSs are described quantitatively using an apparent constant (analogous to *K*_eq_) designated *K*_app_ = [***R***] [***S***]/[***E***] [***N***], where ***S*** is a small molecule thiol released during the ligation process. In addition, the (average) measured differences between the two SSs are characterised using the term Δ*K*_app_, which signifies the extent of the bistability (Δ*K*_app_ = *K*_app_ High SS–*K*_app_ Low SS; Fig. 1d insert). In this study, the values of Δ*K*_app_ were used to elucidate the system’s state in relation to the entire bistable dynamic space. We note that these values can also be useful for future design of functional systems with meaningful signalling.

### Energy-dissipating nature of the bistable network

**Fig. 3:**
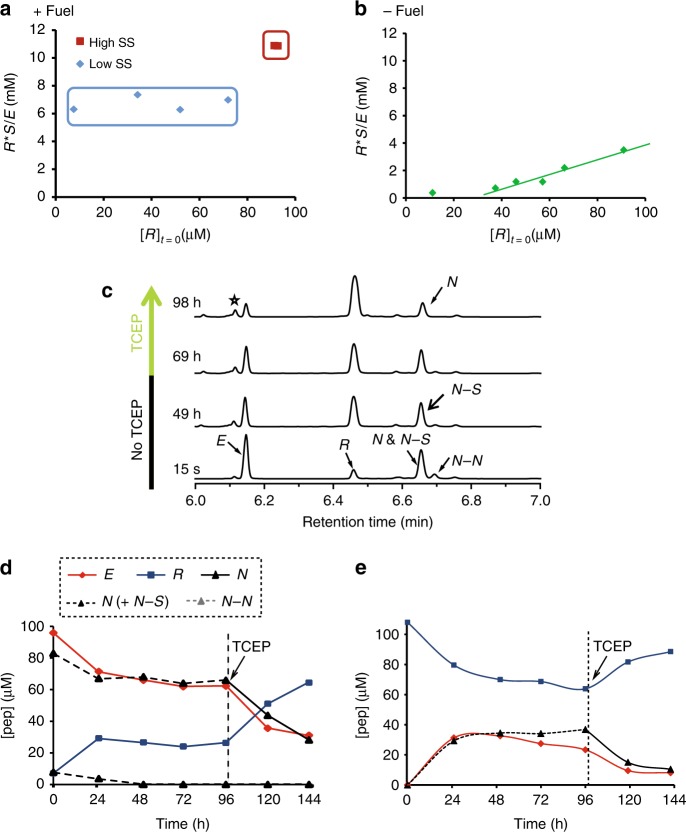
A bistable replication network operating out of equilibrium. Product distribution (R*S/***E***) as function of [***R***] at initiation, for reaction networks operated in the presence (**a**) or absence (**b**) of TCEP as the reducing agent fuelling the reaction. The experimental results used to calculate the product distributions are shown in Supplementary Tables 1 and 3. **c** Time-dependent UPLC chromatograms showing the chemical species formed in a network reaction that was initiated under aerobic conditions (without TCEP) and then reacted by the addition of TCEP (4 mM). **d** Kinetic profile of a network experiment initiated with ***E*** and ***N*** (each 90 ± 5 µM) and ***R*** (10 µM) but without TCEP and then seeded with (10 mM) TCEP after 4 days, and allowed reacting for 2 more days. **e** Kinetic profile of the network reaction initiated with ***R*** (105 µM), without ***E***, ***N*** or TCEP, but seeded with TCEP (10 mM) after 4 days and then allowed reacting for 2 more days. Both reactions in **d** and **e** were carried out in the presence of 10 mM of the thiol ***S*** at 35 °C

Bistable behaviour is a functional product of the network and not a simple reflection of conformational changes of the replicator ***R*** along the reaction coordinate^27^. Therefore, the network reaches either one of the two resting states upon rewiring, depending on the initial concentrations of its components and the environmental conditions applied. At the outset of this study, we realised that a continuously fuelled reducing environment (Fig. 2) is crucial for preventing the bistable system from falling into less defined states, arguably multiple kinetic traps. The ‘engine’ driving the fuelled network is a redox cycle maintained by excess tris(2-carboxyethyl)phosphine hydrochloride (TCEP), which reduces all the unreactive disulphide species into active thiols (***N*** or ***S***) that, in turn, drive several reversible reactions within the autocatalytic network (Fig. 2). To directly study this behaviour, we conducted a set of comparative experiments that revealed the network kinetics and steady-state positions under reduced and oxidised conditions. In the presence of TCEP as the reducing agent, the network reached one of two clearly distinct SSs (+Fuel, Fig. 3a), whereas in the absence of TCEP (−Fuel, Fig. 3b) the same network did not show bistability, but rather the reaction came to an end at multiple resting states, each apparently in linear correlation with the initial set of concentrations.

Further analysis revealed the typical characteristics associated with networks that are kept far from equilibrium by continuous energy consumption. The early-time regimes in Fig. 3c–e (and Supplementary Figs. 5–7) show replication reactions that were initiated under aerobic conditions without TCEP and reached resting states after ~24 h. Disulphide species (N–N and N–S) were formed during this period (Fig. 3c and Supplementary Fig. 5), abolishing their capability to function as nucleophiles (Supplementary Fig. 8). Remarkably, refreshing each of these ‘non-interesting’ resting state mixtures with TCEP (after 60–96 h) with the consequent reduction of the disulphides to thiols (see Supplementary Fig. 5) reactivated the replication process, yielding an appreciable quantity of replicator ***R*** and reaching the expected low (Fig. 3d) and high (Fig. 3e) SS concentrations.

The transient profile of the system’s SSs – a crucial characteristic of the energy-dissipation dependent behaviour – was also clearly established via the following set of control experiments. (i) Network reactions were seeded with TCEP only one time at the initiation (Supplementary Fig. 9). The data shows that after >24 h these reactions reached flat regions in products distribution (supposedly close to the SSs), while later on they reacted to reach other long-lived states. Based on the above discussed data (Fig. 3b), we conclude that after TCEP was exhausted, the network reactions stopped at kinetic traps. (ii) A network reaction was twice seeded with TCEP over the first 24 h (Supplementary Fig. 10), and consequently also reached a resting state close to the SS. At this point, the reaction was followed for a longer time (up to 120 h) without re-fuelling by TCEP, resulting in its halt in a different state, arguably a kinetic trap. Remarkably, re-fuelling with TCEP at 120 h again facilitated reaching the SS position (Supplementary Fig. 10b). (iii) A network reaction was repeatedly seeded with small amounts of TCEP (2 mM; Supplementary Fig. 11). Due to incomplete thiol reduction at each stage, the reaction stopped at different kinetic traps, each time closer to the expected SS. The result of the final (3rd) TCEP seeding step indeed allowed the system to reach a long-lived state very close to the expected SS, as calculated from the respective ‘regular’ experiment with continuous TCEP seeding (Supplementary Table 2, Entry #3).

### Assessment of the bistability parameter space via theory and simulations

**Fig. 4:**
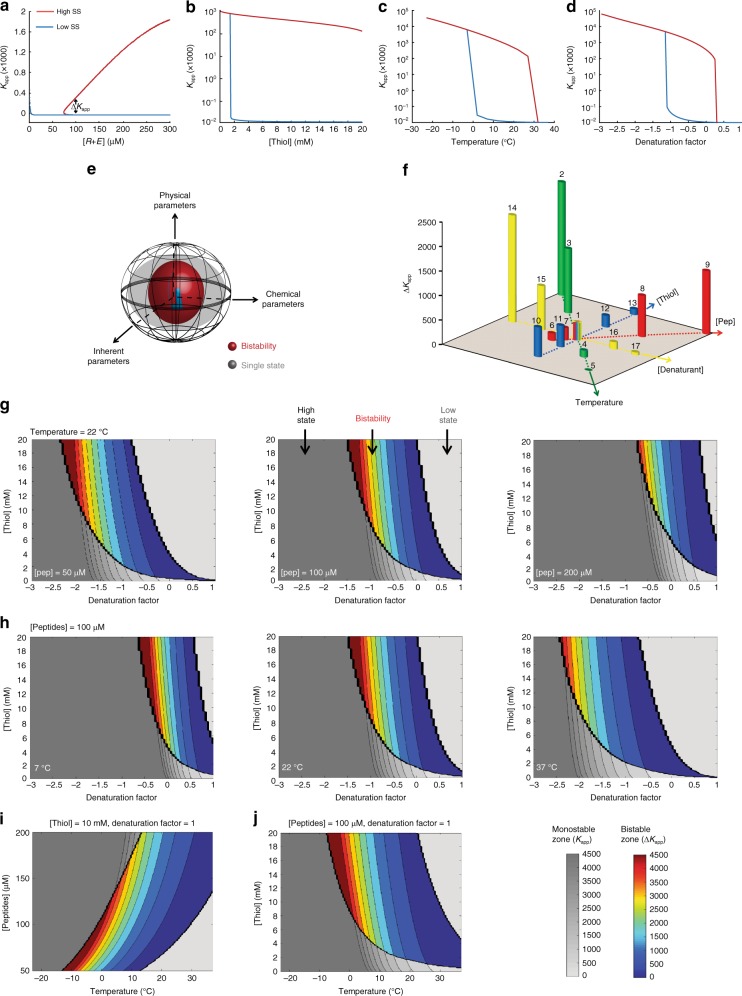
Bistability parameter space revealed by chemical kinetics simulation. **a**–**d** Representative bifurcation diagrams obtained by plotting the calculated *K*_app_ value as a function of the changes in a particular control parameter. **e** General scheme describing the complex parameter space controlling the out-of-chemical-equilibrium replication network. Here, the observed bistable behaviour depends on physical (temperature), chemical (e.g., thiol concentration) and inherent (coiled-coil stability) parameters. **f** Bar graph presenting ∆*K*_app_ as a function of various parameter perturbations. The ∆*K*_app_ value for each case was evaluated for a total peptide concentration of 100 µM, except for the cases studied for the effect of total peptide concentrations (red bars). Numbers above the bars indicate the computation conditions as specified in Supplementary Table 4. The condition set # **1** was considered as the ‘native’ set, with the other cases being presented with respect to **1** (arrows indicate the increase in the applied perturbation, but not the quantitative calibrated change). **g**–**j** Bifurcation contour maps presenting ∆*K*_app_ as a function of combinatorial parameter perturbations. The coloured zone in each panel shows the bistable regime, in which the extent of the bistability (∆*K*_app_) is scaled from blue to red. The *K*_app_ values for single low SSs and single high SSs are shown in grey scale

A multi-dimensional energy space dictates the network behaviour far from equilibrium. Similar to previously studied dynamical systems, we found that the non-linear function characterised here, i.e., bistability, became effective only in sections of this space satisfied by a certain set of parameters (Fig. 4). Within this confined space, bistability is established by non-zero separation between the SSs (Δ*K*_app_ >0), while at the regime edges a sharp phase transition leads to ‘ordinary’ behaviour, in which the system reaches only one SS (Fig. 4). Using the reaction network shown in Fig. 2, we constructed a minimal network reaction model that contains the complete set of respective equations and includes both positive and negative feedback loops (see Section 2, Supplementary Methods). We employed several approaches towards investigating this model. In the first, we deconstructed the model into its two minimal feedback loops and showed mathematically how this leads to either one single SS or three physical SSs (Section 2.1, Supplementary Methods). In the latter case, one of the SSs is unstable, while the other two account for the bistable SSs. A second approach was to run our numerical network simulation, in which we used mass-action kinetics, for specific environmental conditions and specific initial concentrations with the aim to follow the time dependence of the various concentrations. This method has previously been applied by us to observe specific cases leading to bistability^36^. The third approach was to analytically solve for the SSs by mathematically locating the concentrations at which the net process rates were zero. While the first approach gave us a mathematical overview of bistability, and the second approach allowed us to pinpoint individual cases and observe how they lead to bistability, the third approach enabled us to map all the SSs over the entire parameter space and to locate the boundaries of the bistable regime. We therefore followed this approach and computed the rate of each process in Supplementary Equations 2.1–2.8 using mass-action kinetics. We thereby found the global (quadratic) expression for the production of *R* (Eq. 1; for definitions of *g*, *c*, and *T*, see Supplementary Methods section 2.2). The SS was reached when the rate of this reaction was equal to zero.

1 $$\frac{{d[R]}}{{dt}} \approx g\left[ E \right]\left[ N \right] - g\left[ T \right]\left[ S \right] + c\left[ E \right]\left[ N \right]\left[ T \right]^2$$

Figure 4a–d show a selection of computed steady-state *K*_app_ values for the replication network obtained by each time altering one of the critical control parameters, namely, total peptide concentration, thiol concentration, temperature, or denaturant level. Remarkably, triggering the system by changing any one of these factors gave a typical bifurcation diagram, consisting of a middle bistable zone with low (blue) and high (red) SSs, and clear bifurcation points for the phase transitions to regimes of single-low and single-high SSs.

To quantitatively describe the multifaceted space of control parameters affording bistability (Fig. 4e), we constructed a combined two-dimensional bar graph for Δ*K*_app_ as a function of changes in each one of the individual parameters (Fig. 4f). To simplify the data presentation, we selected a ‘native’ middle point (multi-colour bar) pertaining to a network containing 100 μM total peptide and 10 mM thiol concentrations and operating under ambient conditions (T = 22 °C, and denaturant level = 0). The network response to changes in the environment is thus shown with respect to this reference. In addition, we prepared multi-dimensional contour maps of Δ*K*_app_ as a function of several parameters together (Fig. 4g–j). Each contour map displays our numerical results over a three-dimensional parameter space and contains a bistable region. To demonstrate our ability to precisely trace bistability within these parameter space maps, the Δ*K*_app_ heights in the bistable zones (Fig. 4g–j) are emphasised by using colours (scaled from blue to red), while the *K*_app_ values in the single-SS zones are given in grey scale.

The above extensive data set allows us to precisely detect the operational bistable zone and furthermore to draw important conclusions regarding the effect of different parameters and their combinations on the bistability phenomenon. First, we showed that bistability forms under a wide range of parameter combinations. Then, as could be expected (vide infra), we found an increase in the nominal Δ*K*_app_ values for increasing total amounts of peptides in the system. In addition, the vital need for a threshold concentration of thiols as a fuel to drive bistability was evident in all the studied cases, supporting our above-described observation that the system requires a continuous supply of energy. Furthermore, the decrease in Δ*K*_app_ values observed upon raising the temperature or increasing the denaturant concentration demonstrates the need for maintaining the structural integrity of the replicator ***R*** coiled coils, thus facilitating the replication process as a positive feedback that produces the disparity in forward and backward rates leading to bistability. Remarkably, by following the trends in the series of contour maps, e.g., in Fig. 4g, h, we found that changes in several parameters together either compensated for one another, thereby retaining the system’s bistable behaviour, or cancelled one another out, leading to the rapid abolition of bistability.

### Experimental characterisation of the bistability parameter space

**Fig. 5:**
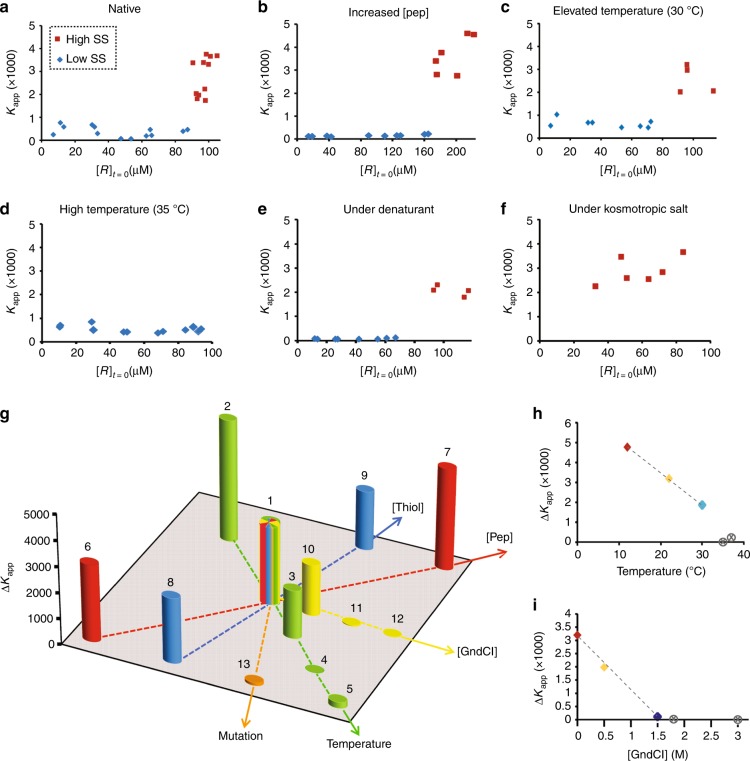
Experimentally investigated bistability parameter space. **a**–**f** *K*_app_ values as a function of the initial concentration of ***R*** for the network studied under various selected conditions, where the reaction initiated with a total peptide concentration [pep] of 100 µM and 500 µM of a small molecule thiol, at room temperature (22 °C), but without a denaturant (GnHCl = 0 M) was considered ‘native’ (**a**); the conditions were changed as follows: increased [pep], i.e., [***R*** + ***E***] = 200 µM (**b**); elevated temperature, T = 30 °C (**c**); high temperature, T = 35 °C (**d**); addition of denaturant, [GnHCl] = 0.5 M (**e**); addition of a kosmotropic salt, [Na_2_SO_4_] = 0.5 M (**f**). **g** Bar graph presenting ∆*K*_app_ as a function of perturbations in the different parameters. The numbers above the bars indicate the experimental conditions specified in Supplementary Table 2. **h** and **i** Quantitative description of ∆*K*_app_ as a function of changes in temperature or GnHCl concentrations. The results show a sharp transition from bistability (∆*K*_app_ >0; coloured data points) to ‘ordinary’ single state (grey points) behaviour. Note that the temperature-dependent and GnHCl-dependent *K*_app_ results are also shown in the supplementary Figs. 34, 35, allowing the detection of hysteresis associated with changing each one of these parameters. All SS concentration results are given in the Supplementary Tables 1 and 2

Using the above quantitative theoretical and simulation analysis as a guide, we then sought to uncover the experimentally operative parameter space. In particular, we probed the effects of changing the system’s inherent parameters, such as total peptide concentration and the type of replicator employed, or manipulating the environmental conditions through changes in temperature or thiol or denaturant (GnHCl) concentrations (Fig. 5 and Supplementary Figs. 16–33). This part of the study again started from the analysis of bistability in a (TCEP fuelled) far-from-equilibrium network under a reasonable ‘native’ set of conditions (Fig. 5a and multi-colour bar in Fig. 5g). Bistability was clearly observed, displaying a switch point from the low state to the high state at about 90μM (i.e., 90%) of the replicator ***R***. Additional experiments (Fig. 5b–f) revealed several characteristic features of the bistable network, in agreement with the simulated data. Increased separation between the low and high SSs, as reflected in an increase in Δ*K*_app_ values, was observed upon an increase in the total peptide concentration (Fig. 5b). A decrease in the Δ*K*_app_ values was observed upon heating the mixture to 30 °C (Fig. 5c) or after adding 0.5 M of GnHCl, the denaturation agent (Fig. 5e). Subsequently, bistability was significantly decreased by employing a full-length mutant thiodepsipeptide that cannot replicate (***R***_**β**_; sequence given in SI)^35^ or by further heating the mixture to 35 °C, yielding a single low SS (Fig. 5d). Furthermore, remarkably, stabilising the coiled-coil assemblies through the addition of a kosmotropic salt ([Na_2_SO_4_] = 0.5 M) led to a single high SS (Fig. 5f). We note that in some cases (e.g., in Fig. 5a, b) a 3^rd^ ‘intermediate’ cluster of SS *K*_app_ values is observed for a narrow range of concentrations residing in between the low and high SSs. In this region, the system is very sensitive to small perturbations in the starting material concentrations and is (evidently) usually slow in reaching the steady state. This behaviour, reflecting a shallower transition between the low and high SSs, has been previously observed in the hysteresis of related experimental bistable systems^22,37^.

A two-dimensional bar graph shows the Δ*K*_app_ values as a function of changes in each of the studied parameters (Fig. 5g), all with high similarity to the simulated network (Fig. 4f). Note that this similarity was applied for a somewhat narrower parameter space than the space we analysed by the simulation, due to obvious physical constraints, such as water freezing at 0 °C, the limited solubility of the peptide and small molecule components of the network, etc. Nevertheless, in addition to finding the Δ*K*_app_ for the network under each of the studied conditions, we were able to experimentally detect the hysteresis (Supplementary Figs. 34, 35), and non-linear phase transition from bistability to a single SS when reaching the temperature limit (Fig. 5h) or the limiting GnHCl concentration (Fig. 5i).

**Fig. 6:**
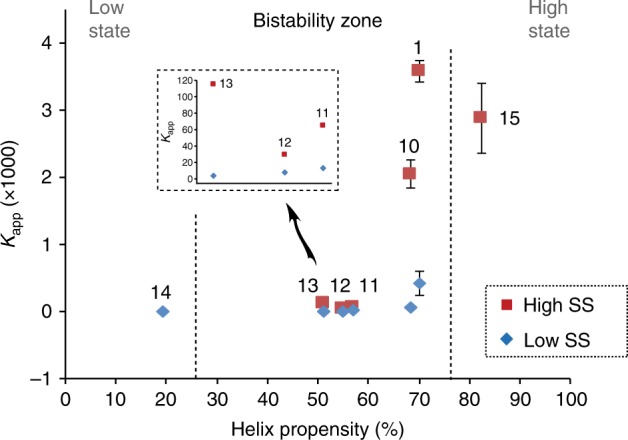
Experimentally derived bifurcation diagram. *K*_app_ values as a function of the thiodepsipeptide coiled-coil propensity coordinate influenced by the type of peptide employed and the added amounts of GnHCl as a denaturant, or of Na_2_SO_4_ as a kosmotropic salt that promotes folding (see Supplementary Fig. 36). Numbers above the data points indicate the experimental conditions specified in Supplementary Table 2; the expanded presentation for data points 11–13 emphasises bistability with small separation between the high and low SSs

Using the data from the entire set of experiments (Supplementary Table 2), we also addressed the challenging goal of reconstructing the entire operative bifurcation diagram—a goal that, to the best of our knowledge, has not been accomplished before. Towards this aim, we followed the network dynamical behaviour in response to changes in various parameters that directly affect the self-assembly process, namely, the amount of denaturant added, the inherent stability of the coiled-coil assembly, and the enhanced assembly effected by adding the kosmotropic salt (see CD data and helix content calculation in Supplementary Fig. 36). Figure 6 follows such a trajectory, displaying a single low SS at unfolding conditions, passing through the middle bistable zone when folding affords efficient replication, and finally again reaching a single state when a too well-folded assembly affords only a single high-concentration SS.

## Discussion

The bistable behaviour demonstrated in this paper expands the repertoire of functions that can be driven by non-enzymatic self-replication, revealing again the potential role of replication for developing smart materials for use in synthetic cells. While assembly of the coiled-coil nanostructure was found to be crucial for driving the replication process, the overall bistability function depended on the delicate interaction between the multiple components of the network. Our combination of theory, simulation and experiment pinpoints the parameters that lead to bistability, including the vital necessity to operate out of chemical equilibrium. Such an analytical approach makes the concept of bistability feasible for previously unreachable reactions, as it can unravel the design rules governing other dynamical synthetic systems, for which information on system kinetics and feedback components is already available. We further propose that quantitative control over bistability, as reflected in our system by Δ*K*_app_, would provide tools to design switchable materials and to devise signal transduction schemes.

## Supplementary information

Supplementary Information

## Data Availability

The data that support the findings of this study are available from the corresponding author upon request.
