# Supplementary material for: A chemically fueled non-enzymatic bistable network
Source: Nat Commun. 2019 Oct 11;10:4636. doi: 10.1038/s41467-019-12645-0 (PMC6789017; doi:10.1038/s41467-019-12645-0)
Supplement: Supplementary file 1 — Supplementary Information [file 41467_2019_12645_MOESM1_ESM.pdf]

Supporting Information

A Chemically Fueled non-Enzymatic Bistable Network

Indrajit Maity, Nathaniel Wagner, Rakesh Mukherjee, Dharm Dev, Enrique Peacock-Lopez, Rivka Cohen-Luria and Gonen Ashkenasy*

* Department of Chemistry, Ben-Gurion University of the Negev, Beer Sheva, 84105, Israel.
Email: gonenash@bgu.ac.il

Table of Contents

Page number

Supplementary Methods.. **2**

1. Experimental Section.. **2**
    1.1 Peptide synthesis and characterization……………………… 2
    1.2 Characterizing the network bistable behaviour……………… 3
2. Theoretical analysis.. **5**
    2.1 Mathematical analysis………………………………………… 5
    2.2 System model ………………………………………………… 8
    2.3 Mathematical derivation and computational procedure……. 9
3. Supplementary Tables……………………………………………… **13**
    Table s1.. 13
    Table s2.. 22
    Table s3.. 23
    Table s4…………………………………………………………………… 24
4. Supplementary Figures…………………………………………….. **25**
    4.1 Energy consuming reaction networks……………………… 25
    4.2 Computed phase diagrams………………………………… 30
    4.3 UPLC Chromatograms of the network reaction at initiation and steady states………………………… 31
    4.4 Networks responses to changes in various control parameters…………………………………… 33
    4.5 Circular dichroism (CD) and helical propensity…………….. 52
5. References……………………………………………………………. **53**

Supplementary Methods

1. Experimental Section

1.1 Peptide synthesis and characterization

All the peptides used in this study have been synthesized on solid phase in our laboratory. The nucleophilic peptides ***N*** and $\boldsymbol{N_\beta}$ were synthesized on a Rink-Amide MBHA (4-methylbenzhydrylamine) resin using standard Fmoc-based chemistry. For ***N*** and $\boldsymbol{N_\beta}$, the Trt-protected thioglycolic acid and Trt-protected 3-mercaptopropionic acids were coupled as the last amino acid, respectively. These peptides were labelled on resin by 4-acetamidobenzoic acid (ABA), incorporated into a specific Lys side chain (Lys-ABA marked in the sequence by X). The standard cocktail for Fmoc chemistry (95% TFA, 2.5% H_2O, 2.5% TIS) was then used for the cleavage and global deprotection of these nucleophile peptides (TFA: Trifluoroacetic acid; TIS : Triisopropylsilane).

The pro-electrophile peptide E-MPA was synthesized by following a modified t-Boc solid phase method on MBHA resin, in which Trt-protected 3-mercaptopropionic acids (3 eq. relative to the resin loading) as a first moiety was coupled to the resin, using (2-(1H-benzotriazol-1-yl)-1,1,3,3-tetramethyluronium hexafluorophosphate) (HBTU) as coupling reagent (3 eq.) and N,N-Diisopropylethylamine (DIPEA) as base (20 eq.) in dimethylformamide (DMF). The Trt group was removed by using a TFA:TIS mixture (95:5) twice (5 and 10 min), and then the synthesis was continued using the standard Boc-chemistry procedure. For the labeling, ABA was coupled as the last moiety of the electrophile sequence. The crude pro-electrophile peptide was obtained after the cleavage and global deprotection with the appropriate cleavage mixture of TFMSA/TFA mixture (TFMSA = trifluoromethanesulfonic acid). The studied electrophile peptide ***E*** (E-MesNa) was synthesized by thiol-thioester exchange reaction from the pro-electrophile E-MPA and the thiol **S** (2-Mercapto-ethanesulfonate sodium salt) in excess amount.

The full-length replicator peptides ***R*** and $\boldsymbol{R_\beta}$ were synthesized by direct ligation. For that the respective thioester peptide (E-MPA, 1 eq.; 3-5 mM) and the corresponding thiol appended nucleophilic peptide (***N*** or $\boldsymbol{N_\beta}$, 1.2 eq.) were dissolved in 1 mL 3-(N-morpholino) propanesulfonic acid (MOPS) buffer (375 mM) at pH ~7.0, containing tris (2-carboxyethyl) phosphine hydrochloride (TCEP) as a reducing agent. The reaction was then allowed to proceed at 37 °C in an incubator for 4-8 hours, until quenched by TFA, and subjected to purification by HPLC.

All the peptides were purified by preparative HPLC using a C18 reverse phase column (Dionex 1100) with a step gradient of solvent A (99% water, 1% acetonitrile (ACN), 0.1% TFA) and B (90% ACN, 10% water, 0.07% TFA). The identity and purity of the peptides were analyzed by HPLC (with the same solvent system for elution), and LCMS. Molecular weights (Mw) observed for all peptides were no more than ±2 off the calculated Mw. Only peptides of 95% purity or higher were used for experiments.

1.2 Characterizing the network bistable behaviour

i) Bistable network characterization

Stock solutions of the peptides (***E***, ***N***, ***R***) were prepared by weighing the lyophilized compounds into 1.5 mL Eppendorf tubes and dissolving in distilled water to yield 0.5 – 1.5 mM solutions. The reactions were carried out in aqueous solutions containing ***E***, ***N***, ***R***, and ***S*** mixtures of variable concentration combinations (Table s1; where for each entry the total amount [***E***] + [***R***] = constant), and with 4 mM of tris (2-carboxyethyl) phosphine hydrochloride (TCEP, except the cases **9, 16, 17** and **18** in which the TCEP concentration was maintained at 12 mM) as a reducing agent and 4-acetamidobenzoic acid (ABA, 50 μM) as an internal standard. Prior to the reaction, the mixtures were equilibrated for 30 minutes under the acidic conditions. Right after that, the reactions were initiated by the addition of 375 mM MOPS buffer at pH ~7, yielding a total volume of 40-80 μL as per experimental requirements. The reactions in Eppendorf tubes were floated in a Julabo chiller to control the reaction temperature. 0.12-0.16 μL of 1.1 M TCEP was continuously re-supplied in the system with 24 h intervals. Aliquots (5-10 μL) were removed at various time points, immediately quenched in 20% glacial acetic acid mixture in water, and stored frozen until subjected to RP-UPLC analysis. The steady state (SS) concentration of the replicator ***R*** and its precursors ***E*** and ***N*** were calculated by averaging the final concentrations at three different times after SS has been reached. The apparent steady state values (K_{app}) were calculated by using K_{app} = ([R][S]/[E][N]) where ***S*** stands for the small molecule thiol concentration. The average high K_{app} and average low K_{app} values were estimated by typically averaging the four highest K_{app} values and four lowest K_{app} values. ΔK_{app} values reflect the difference between the two observed steady state K_{app} values (ΔK_{app} = K_{app} High SS – K_{app} Low SS; Table s2).

ii) Analyzing the fuel dependent behaviour of the bistable networks

In the experiments described in (i), bistability was observed when the fuel molecule TCEP was continuously supplied in 24 h intervals. Therefore, to highlight the effect of

TCEP chemical energy on the bistable behaviour, we have first performed several network reactions (under different sets of conditions) without TCEP, and calculated the apparent resting state constants using the equation as ([***R***][***S***]/[***E***]). The [***N***] was not considered in these cases, because ***N*** was transformed almost completely into ***N-S*** (Table s3). Next, we have repeated the same experiments with several concentration combinations of ***E, N, R*** without TCEP, but then, at a certain time (day 3-4), TCEP was introduced into the mixture to refresh the reaction networks. We have further followed these reactions for 2-3 more days. In addition, in order to probe the transient nature of the system SSs, we have performed similar experiments with varying modes of TCEP administration (see Fig. 9 – 11 and captions).

iii) Disclosing the network response to changes in control parameters

The network experiments described in (i) were repeated multiple times by varying the experimental and environmental conditions (see Table s1). We have assigned the experimental 'native' conditions (case **1**) for the bistable network reaction studied at temperature = 22 °C, [total peptides] = 100 μM, [thiol] = 500 μM, [denaturant] = 0 M. To understand the effect of heat energy on the bistability (cases **2-5**), we carried out the experiments at several temperatures ranging from 12 to 37 °C where all other parameters were kept unchanged. To understand the effect of [peptide] on bistability (cases **6,7**), we carried out the reactions with various concentrations of total peptide materials ranging from 50 to 200 μM, while keeping the other parameters constant. Similarly, we have varied the concentration of small thiol molecule in the reactions (cases **8,9**) from 0.1 to 10 mM, while keeping the other parameters fixed. To understand the effect of denaturant on the network kinetics, we introduced different amounts of the denaturant guanidine hydrochloride (GnHCl). A stock solution of MOPS buffer (375 mM, pH 7) and 6M of GnHCl was utilized, and the reactions were performed with varying the concentration of GnHCl (cases **10-12** and **14**), from 0 to 3 M while other parameters were kept constant. To study the mutation effect, the network reaction was assayed with ***E*** and the mutated analogs $\boldsymbol{R_{\beta}}$, $\boldsymbol{N_{\beta}}$ (case **13**). To investigate the effect of kosmotropic salt (case **15**), the reaction was performed with 0.5M of Na_2SO_4 in otherwise the native condition. In cases of **16-18** both the temperature and concentrations of thiol were varied relative to the native conditions.

2. Theoretical analysis:

In order to keep the system's description analogous to our previous analysis of dynamic non-enzymatic chemical networks,[1-3] the monomeric replicator is defined here by *T* while the total amount of replicator in the system is expressed by *R* (see also below in Supplementary Eq. 2.9). *E*, *N* and *S* are defined as electrophilic and nucleophilic peptides, and small thiol molecules respectively.

2.1 Mathematical analysis

In order to get a better grasp on the mathematics of our network, we can describe the self-replicating peptide system using the following minimal model, which consists of two feedback loops:

Positive Feedback:

$$E + N + T + T \overset{k_1}{\rightarrow} T + T + T + S \tag{1.1}$$

Negative Feedback:

$$T + S \underset{k_2}{\overset{k_{-2}}{\rightleftarrows}} E + N \tag{1.2}$$

The positive feedback loop operates via the formation of the duplex *TT*, which efficiently catalyzes the formation of *T*. In the case of initial low concentration of *T*, the negative feedback loop dominates because of the relatively high concentration of *S*. The concentration of *T* is low compared with *E* and *N*, and there is not enough material to reach a concentration of *TT* to catalyze the formation of *T*. This keeps the concentrations of *E* and *N* relatively high. In contrast, at high initial concentration of *T*, there is enough material to favor the formation of the duplex *TT*, which then efficiently catalyzes the formation of *T*, keeping the concentrations of *E* and *N* relatively lower than *T*.

We now consider only *E*, *N* and *T* as our dynamic variables, as we have shown elsewhere how the intermediate concentrations are totally dependent on these species. Furthermore, we can assume [*E*] = [*N*] without loss of generality. (A more rigorous derivation[4] uses reduced variables, *X* = ([*E*] + [*N*]) / 2 and *Y* = ([*E*] + [*N*]) / 2, leading to the same results.) This leads us to two differential equations:

$$\frac{d[E]}{dt} = -k_1[E]^2[T]^2 - k_2[E]^2 + k_{-2}[T][S] \quad (1.3)$$

$$\frac{d[T]}{dt} = k_1[E]^2[T]^2 + k_2[E]^2 - k_{-2}[T][S] \quad (1.4)$$

Note that $E(t) + T(t) = E(0) + T(0) = W = constant$, since the total material is conserved. Expressing

$E(t) = W - T(t)$, we get the following simple algebraic equation for the steady state:

$$k_1(W-T)^2T^2 + k_2(W-T)^2 - k_{-2}ST = 0 \quad (1.5)$$

Consolidating terms, we get:

$$k_1T^4 - 2k_1WT^3 + (k_1W^2 + k_2)T^2 - (2k_2W + k_{-2}S)T + k_2W^2 = 0 \quad (1.6)$$

This is a quartic equation which has up to four unique solutions for T. It can be shown, however, that one of them corresponds to $T > W$, which is unphysical, leaving us with no more than three unique physical solutions. In the case of bistability, we have two stable and one unstable solutions; otherwise, we have only one physical solution.

The graph below shows the steady state solutions for T as a function of $k_{-2}S$, for a representative numerical example: $W = 7$; $k_1 = 1$; $k_2 = 1/10$. $k_{-2}S$ is used here as a control parameter, because k_1, k_2 and k_{-2} are intrinsic parameters that are set by the microscopic interactions of the peptides, while W, the total amount of material, and S, the thiol concentration, are external parameters that can be controlled macroscopically. W, however, does not affect the internal kinetics, while S, combined with k_{-2}, acts as a kinetic parameter. Supplementary Figure 1a shows that there is always one unphysical solution ($T > W$), and three regions for the physical solutions: (1) a region with only one relatively high SS; (2) a region with three SSs - two stable, one unstable - corresponding to bistability; (3) a region with only one low solution.

To further explore the role of the feedback and the relationship between the positive and negative feedbacks, we can modify the positive feedback by changing k_1 and varying the thiol concentration, or we can fix the thiol concentration and vary k_1. For example, in Supplementary Figure 1b we have increased the value of k_1, and note the changes in

the critical values of $k_{-2}S$ that bring on bistability. In Supplementary Figures 1c and 1d we have fixed $k_{-2}S$ and have varied k_1, again showing how the onset of bistability is critically dependent on the balance between the positive and negative feedbacks.

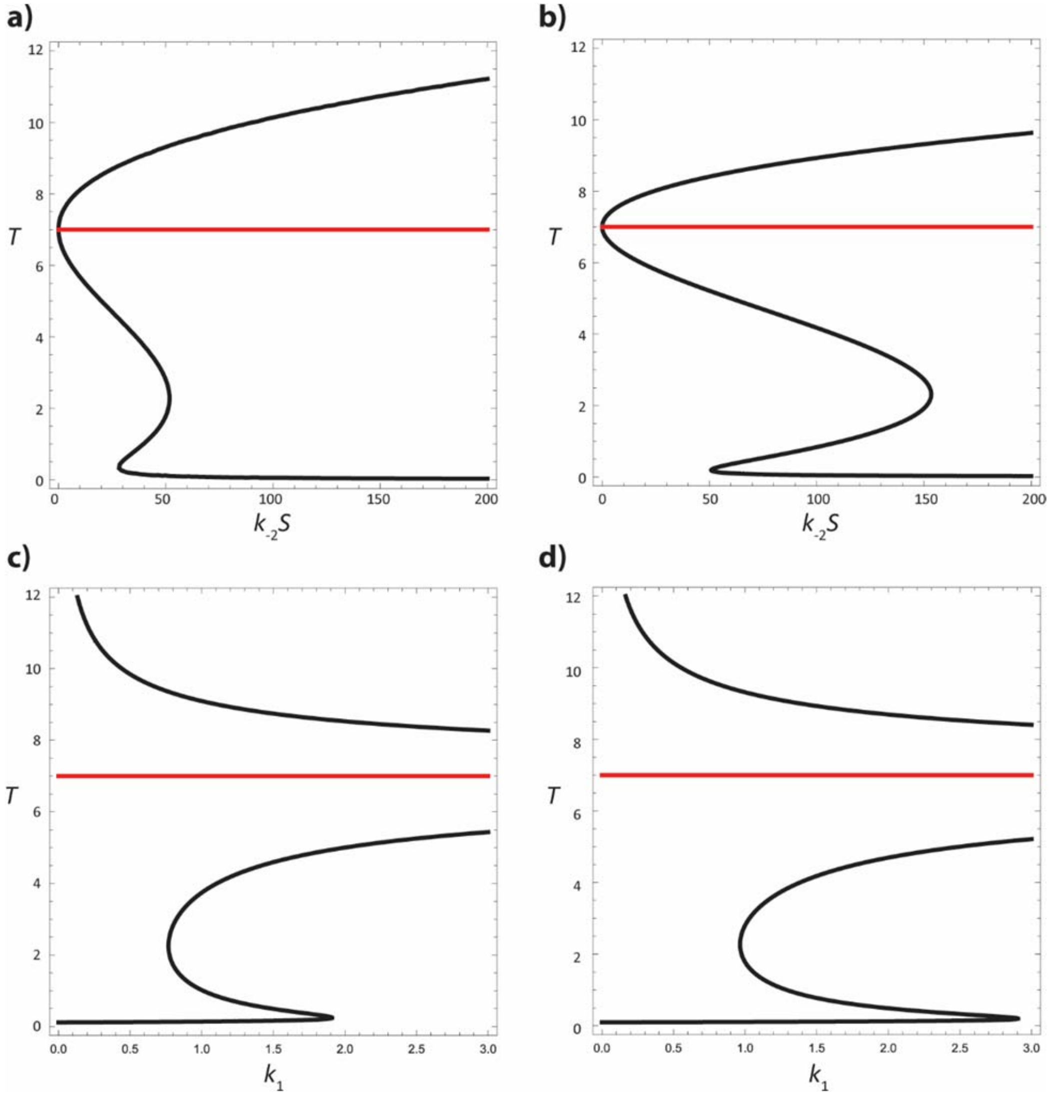

Supplementary Figure 1. Steady state (T) as a function of $k_{-2}S$ (a, b), reference parameters applied: k_1 = 1, W = 7, k_2 = 1/10 (a), and k_1 = 3, W = 7, k_2 = 1/10 (b). Steady state (T) as a function of k_1 (c, d), reference parameters applied: $k_{-2}S$ = 40, W = 7, k_2 = 1/10 (c), and $k_{-2}S$ = 50, W = 7, k_2 = 1/10 (d).

2.2 System model

We model our system with the following mechanisms:

$$E + N \underset{<g>}{\overset{g}{\rightleftarrows}} T + S \tag{2.1}$$

$$E + N + TT \underset{<a>}{\overset{a}{\rightleftarrows}} ENTT \underset{<b>}{\overset{b}{\rightleftarrows}} TTT + S \tag{2.2}$$

$$TTT \underset{<f>}{\overset{f}{\rightleftarrows}} T + TT \underset{<d>}{\overset{d}{\rightleftarrows}} T + T + T \tag{2.3}$$

In addition, the following "harmful" side reactions[3] take place:

$$E + H_2 0 \rightarrow E_{-} \tag{2.4}$$

$$N + N \rightleftarrows N - N \tag{2.5}$$

$$N + S \rightleftarrows N - S \tag{2.6}$$

$$S + S \rightleftarrows S - S \tag{2.7}$$

However, by constantly keeping high concentrations of the reducing agent *TCEP* we can minimize and effectively neutralize these side reactions, and so our model ignores these side reactions. Thus our experimental system, where *TCEP* is continuously pumped in, can be modeled as a quasi-closed system, with corresponding rate constants: *a*, <*d*> and <*f*> for the diffusion processes leading to the formation of the intermediates *ENTT*, *TT* and *TTT*, respectively; <*a*>, *d* and *f* for the respective dissociation of these intermediates; *g* and *b* for the ligation reactions, either directly (via the background reaction) or via autocatalysis; and <*g*> and <*b*> for the reverse ligation reactions. Note that modeling as a quasi-closed system also allows us to set <*b*> ≈ 0, since the *TTT* trimer is much more stable than the *T* monomer in the reduced environment.

Altogether, the system follows the progression of

$$E + N \rightleftarrows R + S \tag{2.8}$$

where the experimentally measured total concentration of reversible template is given by:

$$[R] = [T\ ] + 2\,[ENTT] + 2\,[TT\ \ ] + 3\,[TTT] \qquad (2.9)$$

In this case, the concentration of replicator *R* is equivalent to the experimentally measured total amount of template (*T*).

Using the differential equations (2.10-2.16; derived using mass-action kinetics from Supplementary Equations 2.1-2.3) and the network simulation[3], we can use this model to observe the time progression of our system for specific cases. For example, for the "native" case (Table s4 below, case #1), we observed two steady states: when *R* begins "low" we converge to a low steady state, and when *R* begins "high" we converge to a high steady state, as clearly seen in Supplementary Figure 2.

$$d[E]/dt = -\,g\,[E]\,[N] + \langle g\rangle\,[T]\,[S] - a\,[E]\,[N]\,[TT] + \langle a\rangle\,[ENTT] \qquad (2.10)$$

$$d[N]/dt = -\,g\,[E]\,[N] + \langle g\rangle\,[T]\,[S] - a\,[E]\,[N]\,[TT] + \langle a\rangle\,[ENTT] \qquad (2.11)$$

$$d[T]/dt = g\,[E]\,[N] - \langle g\rangle\,[T]\,[S] + f\,[TTT] - \langle f\rangle\,[T]\,[TT] + 2d\,[TT] - 2\langle d\rangle\,[T]^2 \qquad (2.12)$$

$$d[S]/dt = g\,[E]\,[N] - \langle g\rangle\,[T]\,[S] + b\,[ENTT] - \langle b\rangle\,[TTT]\,[S] \qquad (2.13)$$

$$d[TT]/dt = -\,a\,[E]\,[N]\,[TT] + \langle a\rangle\,[ENTT] + f\,[TTT] - \langle f\rangle\,[T]\,[TT] - d\,[TT] + \langle d\rangle\,[T]^2 \qquad (2.14)$$

$$d[TTT]/dt = b\,[ENTT] - \langle b\rangle\,[TTT]\,[S] - f\,[TTT] + \langle f\rangle\,[T]\,[TT] \qquad (2.15)$$

$$d[ENTT]/dt = a\,[E]\,[N]\,[TT] - (\langle a\rangle + b)\,[ENTT] + \langle b\rangle\,[TTT]\,[S] \qquad (2.16)$$

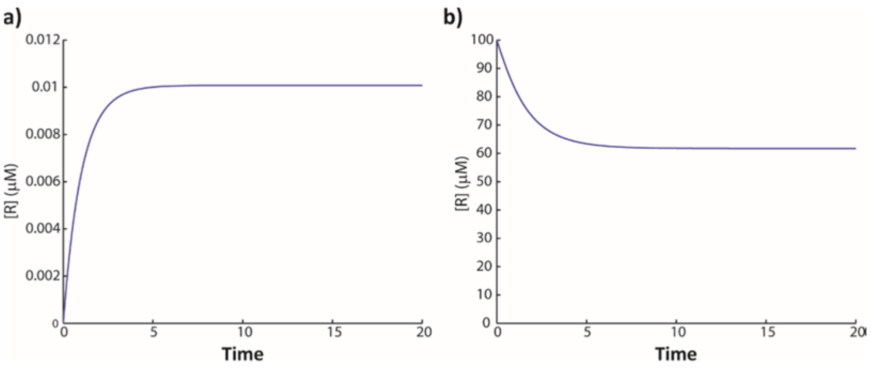

Supplementary Figure 2. Time progression of the network reaction, computed using the numerical network simulation of the model, for the "native" case with initial [*R*] = 0 (a) or initial [*R*] = 100 μM (b). Note the different scale used in (a) and (b).

2.3 Mathematical derivation and computational procedure:

Since our goal is to systematically find the steady state behaviour over a wide range of cases, we can use the rate equations to mathematically solve for the concentrations where the net kinetic processes are zero. Since there is no general closed form mathematical solution for this, we use the following converging iterative procedure.

The rate equations yield the following dependencies for the intermediates:

$$[ENTT]=\frac{a}{\langle a\rangle+b}[E][N][TT]$$

$$[TTT]=\frac{b[ENTT]+\langle f\rangle[T][TT]}{f}$$

$$[TT]=[T]^2\langle d\rangle/d$$

Zeroing the rate of production of *R* yields:

$$\dot{R}=g[E][N]-\langle g\rangle[T][S]+b[ENTT]=g[E][N]-\langle g\rangle[T][S]+c[E][N][T]^2=0 \quad , \quad c=\frac{ab\langle d\rangle/d}{\langle a\rangle+b}$$

As a first approximation, we assume low concentrations for *ENTT* and *TTT*, yielding:

$$[E]\approx[e] \quad [N]\approx[n]$$

$$[T]\approx[R]-2[TT]=\frac{1}{4}\left(\sqrt{\left(\frac{d}{<d>}\right)^2+8\left(\frac{d}{<d>}\right)[R]}-\left(\frac{d}{<d>}\right)\right)$$

Here, [*e*] and [*n*] are the total concentations of electrophile and nucleophile, i.e., [*e*] = [*E*] + [*ENTT*] and [*n*] = [*N*] + [*ENTT*].

Solving for *TT*, *ENTT* and *TTT* yields:

$$[TT]=[T]^2\langle d\rangle/d \qquad [ENTT]=\frac{a}{\langle a\rangle+b}[E][N][TT] \qquad [TTT]=\frac{b[ENTT]+\langle f\rangle[T][TT]}{f}$$

The concentrations of *E*, *N* and *T* can now be calculated to a better approximation:

$$[E]=[e]-[ENTT] \quad [N]=[n]-[ENTT]$$

$$[T]=\frac{1}{4}\left(\sqrt{\left(\frac{d}{<d>}\right)^2+8\left(\frac{d}{<d>}\right)([R]-2[ENTT]-3[TTT])}-\left(\frac{d}{<d>}\right)\right)$$

The above computations for *TT*, *ENTT*, *TTT*, *E*, *N* and *T* are then repeated in order to improve the accuracy, and subsequently $\dot{R}$ is then calculated.

Using this iterative procedure for the "native" case example above, we get the dependence of the rate $\dot{R}$ on [*R*], as shown in Supplementary Figure 3. There are three points where $\dot{R}$ = 0, corresponding to the two stable steady states and the one unstable

steady state described above. Depending on the initial concentation of [*R*] - either below or above the unstable steady state - the system will converge to either the lower or the higher steady state.

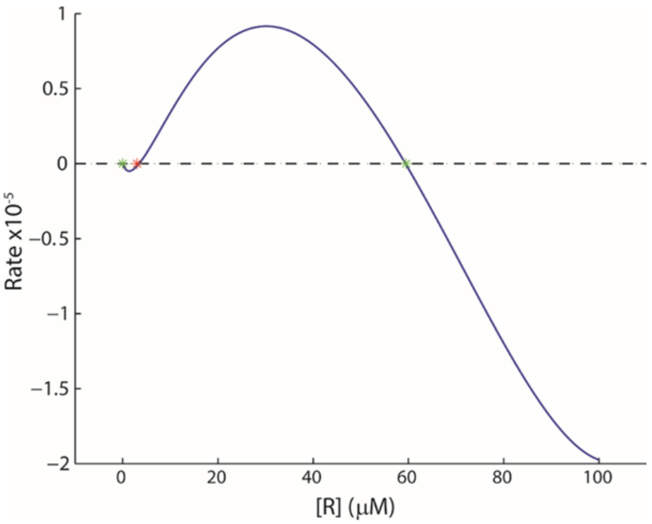

Supplementary Figure 3. Rate ($\dot{R}$) computed as a function of *R* concentration. The three points where $\dot{R} = 0$, correspond to the two stable steady states (green stars) and the one unstable steady state (red star). If the initial concentration of *R* is less than the unstable steady state, the system will converge to the lower stable steady state; if the initial concentration of *R* is greater than the unstable steady state, the system will converge to the higher stable steady state.

This procedure allows us to compute $\dot{R}$ for any set of parameters and initial conditions, and thus locate all cases of $\dot{R} = 0$. The Jacobian $J = \frac{\partial \dot{R}}{\partial [R]}$ can also be computed, enabling us to systematically identify the stable ($J < 0$), as opposed to the unstable ($J > 0$), steady states. Using a computational resolution of 10^{-7}, the above procedure was used to systematically map the extent and magnitude of the bistability - by locating all stable steady states over the entire parameter space, and computing their differences in terms of [*R*] or K_{app} - as presented throughout the paper.

The parameters used to compute the chemical system dynamics were taken similarly to our previously described experimental and simulated networks.[5] The rate constants under varying environmental conditions were chosen according to the guidelines shown in Supplementary Figure 4 and specified in Table s4. We note that in order to choose these parameters, we often used qualitative rules of thumb practiced in the biochemistry

field; alternatively, some of the global changes made (e.g., flat multiplication of all rate constants) might be fine-tuned by adjusting individual parameters. In brief, the parameters for the ‘native’ environmental conditions closely corresponded to our previously studied experimental and simulated systems, typically operating at room temperature (22°C) and pH 7. For higher temperatures (+10°C), we assume a flat two-fold increase in all rate constants of dissociation and ligation (*<a>, d, f, g, b, <g>*), while for lower temperatures (-10°C) we assume a two-fold decrease in those rate constants (factor c_1 in the panel). In addition, we assume a much more moderate temperature dependence for the diffusion rate constants (*a, <d>, <f>*), namely the ratio of the Kelvin temperatures, as described in the panel by factor c_2. Changes in GnHCl (denaturant) concentrations relative to the "native" concentrations give the "denaturation factor" (c_3 in the panel), and we assume a proportional increase in the rate constants of dissociation (*<a>, d, f*).

_a_ , _<d>_ , _<f>_ Diffusion constants (very high)

_g_ , _b_ , _<g>_ Ligation constants

_<a>_ , _d_ , _f_ Dissociation constants

For Temperature ± 10°C: $c_1 = 2^{\pm 1}$ **and** $c_2 = T(°K) / T_0(°K)$

$\Rightarrow \{ <a>, d, f, g, b, <g> \} * c_1 ; \quad \{ a, <d>, <f> \} * c_2$

For the denaturant factor GnHCl $* c_3$

$\Rightarrow \{ <a>, d, f \} * c_3$

Supllementary Figure 4. Rate constants and parameters applied in the reaction network simulation.

3. Supplementary Tables

Supplementary Table 1: Network reaction experiments and the K_{app} values

Case-Entry	[1]Conditions m, t, s, g, r	Parameter Varies from native condition	[2]$[R]_{t=0}$ (μM)	[2]$[E]_{t=0}$ (μM)	[2]$[N]_{t=0}$ (μM)	[3]$[R]_{ss}$ (μM)	[3]$[E]_{ss}$ (μM)	[3]$[N]_{ss}$ (μM)	$[S]_{ss}$ (μM)	K_{app}
**1**-1			90.5	0	0	80.9	3.4	4.0	580. 9	**3460**
**1**-2			98.6	0	0	92.7	3.8	3.9	592.7	**3710**
**1**-3			105	0	0	95.5	3.4	4.6	595.5	**3640**
**1**-4			101	1.5	1.8	87	3.7	3.8	587	**3630**
**1**-5			99.9	0	0	91.8	3.7	4.4	591.8	**3340**
**1**-6			96.9	2.2	1.7	94	3.4	4.9	594	**3350**
**1**-7		**Experimental Native**	97.7	3.6	2.1	93.5	5.9	4.2	593.5	**2240**
**1**-8			98.0	5.6	4.6	92.0	5.8	5.4	592.0	**1740**
**1**-9			93.0	5.5	5.0	85.1	5.4	5.0	585.1	**1840**
**1**-10			94	10.4	12.8	105.5	4.3	7.6	605.5	**1960**
**1**-11	100, 22, 500, 0, R		92.5	10	13.2	101.0	3.7	8.1	601.0	**2030**
**1**-12			87.3	15.0	15.8	78.2	8	12.2	578.2	**463**
**1**-13			84.7	13.7	13.0	83.3	10.0	12.3	583.3	**395**
**1**-14			65	38.5	36	80.3	9.4	10.7	580.3	**463**
**1**-15			65.8	26.7	27.3	83.9	12.8	17.7	583.9	**216**
**1**-16			62.6	29	28.8	76.5	14.5	15.9	576.5	**191**
**1**-17			31.7	69	83	85.6	3.5	24.6	585.6	**582**
**1**-18			30.3	73	84	87.6	3.2	23.9	587.6	**673**
**1**-19			33.5	68.7	63	76.3	11.9	12.4	576. 3	**298**
**1**-20			11.3	90.3	98.3	87.4	2.9	22.8	587.4	**776**
**1**-21			13.3	91.9	105	89.8	3.4	26.8	589.8	**581**
**1**-22			7	94	83	73.6	12.7	13.4	573.6	**248**
**1**-23			47.5	57.6	51.6	76	24.1	25.4	576	**71**
**1**-24			53.2	67	62.7	85.6	26.3	28.7	585.6	**66**
**2**-25	100, **12**, 500, 0, R		100.3	0	0	97.4	2.5	4.8	597.4	**4850**

**Case-Entry**	[1]**Conditions m, t, s, g, r**	**Parameter Varies from native condition**	[2]**$[R]_{t=0}$ (μM)**	[2]**$[E]_{t=0}$ (μM)**	[2]**$[N]_{t=0}$ (μM)**	[3]**$[R]_{ss}$ (μM)**	[3]**$[E]_{ss}$ (μM)**	[3]**$[N]_{ss}$ (μM)**	**$[S]_{ss}$ (μM)**	**K_{app}**
**2**-26			94.6	0	0	91.6	3.1	4.2	591.6	**4160**
**2**-27			102	0	0	93.7	3.7	2.9	593.7	**5180**
**2**-28			105.6	0.8	1.5	99.8	4.6	3.6	599.8	**3610**
**2**-29			92.3	1	1.2	93.4	2.8	3.3	593.4	**6000**
**2**-30			96.5	10.4	10.2	102.2	4.2	5.6	602.2	**2620**
**2**-31	100, **12**, 500, 0, R		95.2	14	10.3	103.3	5.7	4.7	603.3	**2330**
**2**-32		**Temperature**	70	41.8	42.4	89.4	12.8	16.7	589.4	**246**
**2**-33			60.8	42.6	38.2	78.3	17	14.9	578.3	**179**
**2**-34			34	70.7	65	82.3	10.9	17.0	582.3	**259**
**2**-35			31.9	88	76.3	93.0	14.4	12.5	593.0	**306**
**2**-36			11.6	87	91.2	74.5	9.5	22.3	574.5	**202**
**2**-37			11.8	103	92.2	84.7	13.3	20.0	584.7	**186**
**3**-38			96	0	0	90.6	4.5	4.0	590.6	**2970**
**3**-39			95.8	0	0	91	3.7	4.5	591	**3230**
**3**-40			113	4.6	12	88.0	5.6	4.5	588.0	**2050**
**3**-41			91.5	10	9.8	87.8	4.3	6	587.8	**1990**
**3**-42			35.5	74.4	65.7	87.2	6.8	11.4	587.2	**660**
**3**-43	100, **30**, 500, 0, R		33.0	68.1	60	85.0	7	10.9	585.0	**652**
**3**-44			11.1	96.5	89	91.7	5.2	10.5	591.7	**994**
**3**-45			7.6	86.2	82.5	80.6	7.4	11.0	582.6	**591**
**3**-46			72	17.5	20.2	78.3	6.5	9.9	578.3	**704**
**3**-47			70.5	22.8	19.9	79.1	9.4	10.2	579.1	**478**
**3**-48			52	43	46.3	75.1	6.9	13	575.1	**481**
**3**-49			66.1	31.4	29.7	59	7.5	8.3	559	**530**
**4**-50	100, **35**, 500, 0, R		92	0	0	70.8	7.1	12.4	570.8	**459**

Case-Entry	[1]Conditions m, t, s, g, r	Parameter Varies from native condition	[2]$[R]_{t=0}$ (μM)	[2]$[E]_{t=0}$ (μM)	[2]$[N]_{t=0}$ (μM)	[3]$[R]_{ss}$ (μM)	[3]$[E]_{ss}$ (μM)	[3]$[N]_{ss}$ (μM)	$[S]_{ss}$ (μM)	K_{app}
**4**-51			93.4	0	0	72	6.2	12	572	**554**
**4**-52			84.2	7.5	5.3	71.1	6.3	12.8	571.1	**504**
**4**-53	100, **35**, 500, 0, R		89	7.4	5.3	72.6	5.8	11.1	572.6	**646**
**4**-54			71	32.2	34.5	68.4	8.4	10.5	568.4	**441**
**4**-55			68	32.7	33	70	9.9	10.2	570	**395**
**4**-56			50	55	57	73	9	10.9	573	**424**
**4**-57		**Temperature**	48	53	57.4	71	8.5	11.4	571	**416**
**4**-58			29	78	77	75.9	4.5	11.5	575.9	**845**
**4**-59			30.3	71.6	73.6	70.3	5.4	14.5	570.3	**512**
**4**-60			10.6	93	89	69.2	4.6	12.4	569.2	**691**
**4**-61			10.2	90.7	88.3	69.3	5	12.4	569.3	**636**
**5**-62			102.6	0	0	74.8	13.3	10	574.8	**323**
**5**-63			106	0	0	79.4	13.9	11.7	579.4	**283**
**5**-64			95.5	2.5	7.5	71.8	14.3	13.5	571.8	**213**
**5**-65	100, **37**, 500, 0, R		90.4	1.0	4.3	56.5	14.7	14.6	556.5	**146**
**5**-66			88.7	5.6	7.5	62.6	15	9.8	562.6	**239**
**5**-67			85.1	0.5	4	50	12.4	11.3	550	**196**
**5**-68			33.4	69.4	68.0	46.9	24.1	26	546.9	**41**
**5**-69			25.2	64.9	62.6	42.4	23.7	25.2	542.4	**38**
**5**-70			11.3	96.6	82.5	41.8	27.8	27.6	541.8	**29**
**5**-71			7.8	91.1	80.6	36.2	27.4	24.7	536.2	**29**
**6**-72			49	0	0	46.6	3.5	2.6	546.6	**2800**
**6**-73			56	0	0	52.4	3.3	2.9	552.4	**3030**
**6**-74	**50**, 22, 500, 0, R	**[Peptides]**	52.5	1.5	0.6	44.8	2.6	3.5	544.8	**2680**
**6**-75			50.1	2.0	1.8	43.7	2.7	2.4	543.7	**3670**

Case-Entry	[1]Conditions m, t, s, g, r	Parameter Varies from native condition	[2]$[R]_{t=0}$ (μM)	[2]$[E]_{t=0}$ (μM)	[2]$[N]_{t=0}$ (μM)	[3]$[R]_{ss}$ (μM)	[3]$[E]_{ss}$ (μM)	[3]$[N]_{ss}$ (μM)	$[S]_{ss}$ (μM)	K_{app}
**6**-76	**50**, 22, 500, 0, R	[Peptides]	39	22.2	17	36.7	12.6	10.5	536.7	**144**
**6**-77			36	22.6	17.2	36.1	12.8	10.7	536.1	**141**
**6**-78			32.9	9.4	10.7	30.7	6.9	10.2	530.7	**231**
**6**-79			27.6	9.9	9.7	36	9.3	10	536	**207**
**6**-80			17.3	32.8	33.1	25.8	5.5	16.5	525.8	**150**
**6**-81			16.6	33.8	33	28.2	14.3	16.7	528.2	**62**
**6**-82			10.8	46	36	25.8	23.8	16.2	525.8	**35**
**6**-83			11	47	36	27.4	18.2	14.3	527.4	**56**
**6**-84			12	45	35	23.9	18	15.1	523.9	**46**
**7**-85	**200**, 22, 500, 0, R		222	0	0	209	5.2	6.3	709	**4520**
**7**-86			213.8	0	0	197.2	5.0	5.9	697.2	**4660**
**7**-87			181.4	1.7	1.3	159	5.0	5.5	659	**3810**
**7**-88			174.6	0	0	151	5.0	5.7	651	**3450**
**7**-89			176	3.5	2.3	165.4	7.0	5.6	665.4	**2810**
**7**-90			201.2	10.7	9.6	185.3	6.7	6.9	685.3	**2750**
**7**-91			165.2	60	54	183.2	25.3	22.7	683.2	**218**
**7**-92			159.8	60.1	54.6	184	26	22.7	684	**213**
**7**-93			130	105.1	89.4	181	34.4	22.7	681	**158**
**7**-94			125	100	92.6	185.6	32.7	24.3	685.6	**160**
**7**-95			110	128.8	123	181.2	38	23.4	681.2	**139**
**7**-96			90	140.5	116	178.3	44.3	17.2	678.3	**159**
**7**-97			43	190	174	166.8	29.8	32.4	666.8	**115**
**7**-98			37.4	187.7	171.4	170.3	26.3	30.6	670.3	**142**
**7**-99			14.2	198	195	162.2	23.9	38.9	662.2	**116**
**7**-100			19	208	204	178.8	24.4	43.4	678.8	**115**

**Case-Entry**	[1]**Conditions m, t, s, g, r**	**Parameter Varies from native condition**	[2]**$[R]_{t=0}$ (μM)**	[2]**$[E]_{t=0}$ (μM)**	[2]**$[N]_{t=0}$ (μM)**	[3]**$[R]_{ss}$ (μM)**	[3]**$[E]_{ss}$ (μM)**	[3]**$[N]_{ss}$ (μM)**	**$[S]_{ss}$ (μM)**	**K_{app}**
**8**-101			96	0	0	94.9	1.9	3.5	194.9	**2780**
**8**-102			103.7	0	0	96.4	1.7	3.3	196.4	**3380**
**8**-103	100, 22, **100**, 0, R		108.5	1.3	2.7	103.6	2.0	4.5	203.6	**2340**
**8**-104			98.2	1.0	2.8	91.4	1.7	4.5	191.4	**2290**
**8**-105			80.1	21.7	23.2	82.6	10.2	13.6	182.6	**109**
**8**-106			76.3	21	22.3	81.4	9	12	181.4	**137**
**8**-107			66	46.8	49.7	81	11	22.4	181	**60**
**8**-108			63.8	45	49.7	86	12.5	22.2	186	**58**
**8**-109			42	84	73.8	96.6	11.3	7.3	196.6	**230**
**8**-110			29.5	80.1	67.6	80.7	17.9	15.8	180.7	**52**
**8**-111			11.2	91.4	93.4	85.9	7.9	16.9	185.9	**120**
**8**-112		**[Thiol]**	8.4	96.6	90.4	79.5	8.0	11.9	179.5	**150**
**9**-113			98.3	0	0	76.3	15.5	17.8	10076.3	**2790**
**9**-114			98.8	0	0	85.5	16.3	17.9	10085.5	**2960**
**9**-115			92	0	0	71.2	15.5	15.5	10071.2	**2990**
**9**-116			102	0	0	77.3	17.9	16.4	10077.3	**2650**
**9**-117			96	7	5.6	84.8	17.8	15.3	10084.8	**3140**
**9**-118			97.4	8.6	5.6	78.1	18.8	15.5	10078.1	**2700**
**9**-119			91	12.8	11.3	82.6	20.6	19.9	10082.6	**2030**
**9**-120	100, 22, **10000**, 0, R		88.8	10.7	10.0	76.7	19.4	18.8	10076.7	**2120**
**9**-121			93.9	10.8	14	84.3	17.7	21.2	10084.3	**2270**
**9**-122			92.3	12	10.8	77.6	19.8	20.2	10077.6	**1960**
**9**-123			67.5	44	36	70.9	34	31.8	10070.9	**660**
**9**-124			55	43.5	37.7	62.8	34.2	32	10062.8	**577**
**9**-125			35.9	70.7	63	68.9	28.0	29.5	10068.9	**840**

Case-Entry	[1]Conditions m, t, s, g, r	Parameter Varies from native condition	[2][R]$_{t=0}$ (μM)	[2][E]$_{t=0}$ (μM)	[2][N]$_{t=0}$ (μM)	[3][R]$_{ss}$ (μM)	[3][E]$_{ss}$ (μM)	[3][N]$_{ss}$ (μM)	[S]$_{ss}$ (μM)	K$_{app}$
**9**-126			33	71.9	63	64.8	29.9	29.2	10064.8	747
**9**-127		**[Thiol]**	29.3	67	63	71.3	29.0	31.7	10071.3	781
**9**-128	100, 22, **10000**, 0, R		15.5	99.3	82.2	63.5	37.7	27	10063.5	628
**9**-129			10.9	97	87.2	60.6	33	36.6	10060.6	505
**9**-130			6	99	82	62.1	31.0	32.9	10062.1	613
**10**-131			96	0	0	86.4	5.2	4.3	586.4	2270
**10**-132			114	1.9	1	107	8.3	4.4	607	1780
**10**-133	100, 22, 500, **0.5**, R		93.3	0	0	86.4	5.5	4.4	586.3	2090
**10**-134			116	1.0	3.8	108	6.5	4.9	608	2060
**10**-135			67	36.7	28.2	74.4	24.4	15.6	574.4	112
**10**-136			61	38.5	30.9	78.5	24.7	19	578.5	97
**10**-137		**[Denaturant]**	55	66	54.7	77.3	31.2	23.6	577.3	61
**10**-138			42	59	45.8	68.6	26.8	22.5	568.6	65
**10**-139			27.7	77	61	59.6	31.9	18.4	559.6	57
**10**-140			26	76	67.8	64.9	28.3	19.2	564.9	67
**10**-141			13.7	96	82.5	70.6	27.7	21.2	570.6	69
**10**-142			13.5	98	80	67.7	31.2	16.7	567.7	74
**11**-143			105	0	0	72.3	27.7	18.8	572.3	79
**11**-144			105	0	0	67.8	29.4	17.6	567.8	74
**11**-145	100, 22, 500, **1.5**, R		92.5	12.1	9.6	63.4	32	17.4	563.4	64
**11**-146			92.2	11.5	9.2	63.3	35.2	23.2	563.3	44
**11**-147			72	30.9	25.9	35.2	49	23.6	535.2	16
**11**-148			45	65.2	50.1	33.6	61.4	30	533.6	10
**11**-149			34	75.8	61	43.1	54.1	28.3	543.1	15
**11**-150			28.3	75.8	62	36.7	52.2	25.6	536.7	15

**Case-Entry**	[1]**Conditions m, t, s, g, r**	**Parameter Varies from native condition**	[2]**$[R]_{t=0}$ (μM)**	[2]**$[E]_{t=0}$ (μM)**	[2]**$[N]_{t=0}$ (μM)**	[3]**$[R]_{ss}$ (μM)**	[3]**$[E]_{ss}$ (μM)**	[3]**$[N]_{ss}$ (μM)**	**$[S]_{ss}$ (μM)**	**K_{app}**
**11**-151			13	97	82	39.5	56.5	25.8	539.5	**15**
**11**-152	100, 22, 500, **1.5**, R		8.2	95.6	87	40.1	51.4	35.23	540.1	**12**
**12**-153			101	0	0	58.9	44.8	27.5	558.9	**27**
**12**-154			94.8	0	0	59	36.4	28.4	559	**32**
**12**-155		**[Denaturant]**	89.6	7.3	3	56.2	39.2	25.4	556.2	**31**
**12**-156	100, 22, 500, **1.8**, R		88	10	5	56.7	38.7	26.7	556.7	**31**
**12**-157			44.2	52.8	51.7	35.4	54.4	40.5	535.4	**9**
**12**-158			42.1	56.6	49.3	40.2	54	39.2	540.2	**10**
**12**-159			30	60.5	60.9	34.1	55.7	36.8	534.1	**9**
**12**-160			24.6	73.5	75.4	34.2	55.7	40.4	534.2	**8**
**14**-161			96	0	0	6.7	96.8	66.5	506.7	**0.5**
**14**-162			97	0	0	8.6	92.9	61.7	508.6	**0.8**
**14**-163			90	29.4	27.6	8.9	107.5	47	508.9	**0.9**
**14**-164	100, 22, 500, **3**, R		80.1	11	4.5	8.9	84.6	57.3	508.9	**0.9**
**14**-165			82.5	5.7	4.3	9.6	77.4	56.9	509.6	**1.1**
**14**-166		**Mutation**	60	62.4	59.3	9	99.2	41.9	509	**1.1**
**14**-167			28	76	71.5	8.6	88.6	63.8	508.6	**0.8**
**14**-168			27.9	74.3	72.8	8.3	87.6	61.7	508.3	**0.8**
**14**-169			10.9	102	94.7	10.1	95	62.2	510.1	**0.9**
**14**-170			8.9	96.8	88.9	8.4	89	59	508.4	**0.8**
**13**-171			102	0	0	77.8	19.8	19	577.8	**119**
**13**-172			104	0	0	76.9	19.3	19.1	576.9	**120**
**13**-173	100, 22, 500, 0, $\mathbf{R_\beta}$		102	2.4	4.2	80.3	20	21	580.3	**111**
**13**-174			103	2.9	4.8	80.9	20.7	21.7	580.9	**105**
**13**-175			88	11.5	12.2	64.5	21.6	19.6	564.5	**86**

Case-Entry	[1]Conditions m, t, s, g, r	Parameter Varies from native condition	[2][R]$_{t=0}$ (μM)	[2][E]$_{t=0}$ (μM)	[2][N]$_{t=0}$ (μM)	[3][R]$_{ss}$ (μM)	[3][E]$_{ss}$ (μM)	[3][N]$_{ss}$ (μM)	[S]$_{ss}$ (μM)	K_{app}
**13**-176			74.8	22.9	19.4	53.7	42	37.6	553.7	**19**
**13**-177		**Mutation**	56	55.4	40.9	48	54	48.7	548	**10**
**13**-178	100, 22, 500, 0, **$R_β$**		36	75.8	59	36.3	70	57.9	536.3	**5**
**13**-179			27	72	74.8	39.3	50.8	58.6	539.3	**7**
**13**-180			26	66	68.9	35.3	48.8	57.9	535.3	**7**
**13**-181			7	84	91.4	22.8	58.4	73.6	522.8	**3**
**13**-182			8.14	90.2	92.8	23	62	72.8	523	**3**
**15**-183			88	9.12	11	84.4	3.0	4.4	584.4	**3740**
**15**-184			72	17	19.4	80.8	4	4.1	580.8	**2860**
**15**-185		Kosmotropic salt	63.8	34.4	34	83.3	4.2	4.5	583.3	**2570**
**15**-186	100, 22, 500, 0, R **(+ 0.5 M Na_2SO_4)**		51.2	46	48.2	89.6	3.5	5.9	589.6	**2560**
**15**-187			47.7	66	63	104.8	5.3	3.4	597.2	**3220**
**15**-188			32.6	75.9	69.2	99.9	6.5	4	599.9	**2310**
**16**-189			99	0	0	83.6	9.3	7.9	10083.6	**11470**
**16**-190		Thiol and Temperature (low)	94	5.2	5.7	87.5	10.9	9.5	10087.5	**8530**
**16**-191			93.8	4.9	5.6	85.6	9.2	9	10085.6	**10430**
**16**-192	100, **12, 10000,** 0,R		86	9.2	9.3	80.9	9.3	10.3	10080.9	**8510**
**16**-193			88.4	11.4	8.7	87.0	13.8	9.6	10087	**6620**
**16**-194			85	34.2	34.4	76.8	41.1	27.4	10076.8	**687**
**16**-195			80	34	31.2	72.5	38.2	23.8	572.5	**803**
**16**-196			57	70	60	70	44.3	38.9	570	**409**
**16**-197			33	78	70	65.1	30.4	31.1	10065.1	**693**
**16**-198			34	76.6	70.3	65.0	30.5	35	10065	**613**
**16**-199			8.8	95	92.3	52.3	40.3	51.4	10052.3	**254**
**16**-200			7.4	82	81	46.7	43.1	56.5	10046.7	**193**

**Case-Entry**	[1]**Conditions m, t, s, g, r**	**Parameter Varies from native condition**	[2]$[R]_{t=0}$ **(μM)**	[2]$[E]_{t=0}$ **(μM)**	[2]$[N]_{t=0}$ **(μM)**	[3]$[R]_{ss}$ **(μM)**	[3]$[E]_{ss}$ **(μM)**	[3]$[N]_{ss}$ **(μM)**	$[S]_{ss}$ **(μM)**	K_{app}
**17**-201			90	1	2	66.4	19.9	21.3	10066.4	**1580**
**17**-202	100, **30, 10000,** 0,R	Thiol and Temperature (high)	96.2	1.5	2.1	72.2	18.4	20.2	10072.1	**1950**
**17**-203			99.6	9.2	12.9	82.4	19.3	22.3	10082.4	**1930**
**17**-204			96	16	10.7	91.2	21.7	17.7	10091.2	**2400**
**17**-205			33	69.3	60	68.6	21.0	23.0	10068.6	**1430**
**17**-206			32.3	71.5	62.6	64	20.5	23.8	10064	**1320**
**17**-207			11	89.7	84.7	65.7	17.2	29.7	10065.7	**1300**
**17**-208			9.8	90.6	79.2	66.7	18.4	26	10066.7	**1400**
**18**-209			94	0	0	55.8	28.9	27.8	10055.8	**698**
**18**-210			90	0	0	50.2	30.5	30.6	10050.2	**541**
**18**-211			86	6.5	7	56.7	31.1	30.4	10056.7	**603**
**18**-212			92	4.8	7	56	33	34	10056	**502**
**18**-213			87	8.3	10	51.5	29.4	29.2	10051.5	**603**
**18**-214	100, **35, 10000,** 0,R	Thiol and Temperature (very high)	85	29.5	21.1	48.2	58.6	42.9	10048.2	**193**
**18**-215			78	26.5	17.1	51	56	45	10051	**203**
**18**-216			65	57.3	41.1	45.8	62.4	52.2	10045.8	**141**
**18**-217			46	72	60	45	61.8	42.3	10045	**173**
**18**-218			47	74	61	41.9	72	51	10041.9	**115**
**18**-219			22	99.1	84.2	37.6	78.1	58.6	10037.6	**82**
**18**-220			20	100	77.1	36.2	76.7	49.7	10036.2	**95**
**18**-221			15	89.8	90	42.1	47.6	58.7	10042.1	**151**

[1]Reaction conditions [m (μM), t (°C), s (μM), g (M), r] refer to the total [peptide], temperature, thiol concentration, denaturant GnHCl concentration and studied replicator, respectively. [2]$[R]_{t=0}$, $[E]_{t=0}$ and $[N]_{t=0}$ indicate initial concentration of replicator, electrophile and nucleophile at t = 0 minutes of the replication reaction. [3]$[R]_{ss}$, $[E]_{ss}$, [N]ss and $[S]_{ss}$ represent the average concentration of replicator, electrophile, nucleophile and small thiol molecule at steady state. Experiments in higher temperature (37 °C) suffer from a low-extent hydrolysis (≤ 20%) of E.

Supplementary Table 2: ΔK_{app} values obtained from experiments under the variable conditions

Case	Conditions m, t, s, g, r	Parameter Varies from native condition	[1]High K_{app}	Std. dev.	[1]Low K_{app}	Std. dev.	Δ K_{app}
1	100, 22, 500, 0, R	Native	3580	157	427	179	3160
2	100, **12**, 500, 0, R	Temperature	4920	982	238	55	4680
3	100, **30**, 500, 0, R		2560	634	724	182	1840
4	100, **35**, 500, 0, R		×	×	544	137	×
5	100, **37**, 500, 0, R		265	49	34	6	231
6	**50**, 22, 500, 0, R	[Peptides]	3040	440	98	55	2950
7	**200**, 22, 500, 0, R		3950	424	122	7	3830
8	100, 22, **100**, 0, R	[Thiol]	2700	503	138	74	2560
9	100, 22,**10000**, 0, R		2950	182	661	127	2280
10	100, 22,500, **0.5**, R	[Denaturant]	2050	202	67	7	1980
11	100, 22,500, **1.5**, R		65	15	14	1	51
12	100, 22,500, **1.8**, R		30	2.3	9	1	21
14	100, 22,500, **3**, R		×	×	0.9	0.2	×
13	100, 22, 500, 0, $\mathbf{R_\beta}$	Mutation	114	7	5	2	109
15	100, 22, 500, 0, R **(+ 0.5 M Na_2SO_4)**	Kosmotropic salt	2880	525	×	×	×
16.	100, **12**,**10000**, 0, R	Thiol and Temperature (low)	9740	1467	438	251	9300
17	100, **30**,**10000**, 0, R	Thiol and Temperature (high)	1960	335	1362	65	602
18	100, **35**,**10000**, 0, R	Thiol and Temperature (very high)	611	65	111	30	500

Note: The high K_{app} and low K_{app} values are calculated by averaging the four highest K_{app} and four lower K_{app} values (from Table s1), respectively. For the cases **4**, **14** and **15** (single steady state) all the data was subjected to averaging.

Supplementary Table 3: Network reaction experiments ran in the absence of the fuel molecule TCEP

Case-Entry	[1]Conditions m, t, s, g, r	Parameter Varies from native condition	[2] $[R]_{t=0}$ (μM)	[2] $[E]_{t=0}$ (μM)	[2] $[N]_{t=0}$ (μM)	[3] $[R]_{ss}$ (μM)	[3] $[E]_{ss}$ (μM)	$[S]_{ss}$ (μM)	R*S/E (mM)
**3a-1**	100, **30**, 500, 0, R	**Without TCEP**	91	28	15	86.3	14.5	586. 3	**3490**
**3a-2**			66.3	33.1	24.6	59.8	15.6	559.8	**2146**
**3a-3**			57.1	55.1	47.2	58	27.5	558	**1177**
**3a-4**			46	55.7	58.4	53.8	25.2	553.8	**1182**
**3a-5**			37.4	75	66	59.7	47	559.7	**711**
**3a-6**			11.2	96.5	79	38	55.3	538	**370**

[1]Reaction conditions [m (μM), t (°C), s (μM), g (M), r] refer to the total [peptide], temperature, thiol concentration, denaturant GnHCl concentration and studied replicator, respectively. [2] $[R]_{t=0}$, $[E]_{t=0}$ and $[N]_{t=0}$ indicate initial concentration of replicator, electrophile and nucleophile at t = 0 minutes of the replication reaction. [3] $[R]_{ss}$, $[E]_{ss}$, and $[S]_{ss}$ represent the average concentration of replicator, electrophile, and small thiol molecule at resting state. The resting state parameters were calculated from the equation as [***R***][***S***]/[***E***]. The [***N***] was not considered in this case because ***N*** was transformed almost completely into ***N-S***.

Supplementary Table 4: Network reaction computations and the resulted ΔK_{app} values

case	Simulated parameters	Corresponding experimental conditions (m, t, s, f_g)	Parameters	ΔK_{app}
**1**	a = 10^9, <d> = <f>= 10^6, <a> = 10, d = 10, f = 1000, g = 1, b = 10, <g> = 100	100, 22, 10000, 1	**Native conditions**	353
**2**	a = 0.95*10^9 <d> = <f> = 0.95*10^6 <a> = 3.5 d = 3.5 f = 350, g = 0.35 b = 3.5 <g> = 35	100, **7**, 10000, 1		2320
**3**	a = 0.97*10^9, <d> = <f>= 0.97*10^6, <a> = 5, d = 5, f = 500, g = 0.5, b = 5, <g> = 50	100, **12**, 10000, 1	**Temperature**	1320
**4**	a = 1.017*10^9, <d> = <f> = 1.017*10^6 , <a> = 14.1, d = 14.1, f = 1410, g = 1.41, b = 14.1, <g> = 141	100, **27**, 10000, 1		138
**5**	a = 1.0310^9 <d> = <f> = 1.0310^6 <a> = 20 d = 20 f = 2000, g = 2 b = 20 <g> = 200	100, **32**, 10000, 1		0
**6**	a = 10^9, <d> = <f>= 10^6, <a> = 10, d = 10, f = 1000, g = 1, b = 10, <g> = 100	**80**, 22, 10000, 1		152
**7**	a = 10^9, <d> = <f>= 10^6, <a> = 10, d = 10, f = 1000, g = 1, b = 10, <g> = 100	**90**, 22, 10000, 1		255
**8**	a = 10^9, <d> = <f>= 10^6, <a> = 10, d = 10, f = 1000, g = 1, b = 10, <g> = 100	**150**, 22, 10000, 1	**[Peptides]**	845
**9**	a = 10^9, <d> = <f>= 10^6, <a> = 10, d = 10, f = 1000, g = 1, b = 10, <g> = 100	**200**, 22, 10000, 1		1290
**10**	a = 10^9, <d> = <f>= 10^6, <a> = 10, d = 10, f = 1000, g = 1, b = 10, <g> = 100	100, 22, **3000**, 1		609
**11**	a = 10^9, <d> = <f>= 10^6, <a> = 10 d = 10 f = 1000, g = 1 b = 10 <g> = 100	100, 22, **7000**, 1		441
**12**	a = 10^9, <d> = <f>= 10^6, <a> = 10 d = 10 f = 1000, g = 1 b = 10 <g> = 100	100, 22, **15000**, 1	**[Thiol]**	240
**13**	a = 10^9, <d> = <f>= 10^6, <a> = 10 d = 10 f = 1000, g = 1 b = 10 <g> = 100	100, 22, **20000**, 1		120
**14**	a = 10^9, <d> = <f>= 10^6, <a> = 2 d = 2 f = 200, g = 1 b = 10 <g> = 100	100, 22, 10000, **0.2**		2180
**15**	a = 10^9, <d> = <f>= 10^6, <a> = 5 d = 5 f = 500, g = 1 b = 10 <g> = 100	100, 22, 10000, **0.5**	**Denaturation factor**	901
**16**	a = 10^9, <d> = <f>= 10^6, <a> = 15 d = 15 f = 1500, g = 1 b = 10 <g> = 100	100, 22, 10000, **1.5**		150
**17**	a = 10^9, <d> = <f>= 10^6, <a> = 19, d = 19, f = 1900, g = 1, b = 10, <g> = 100	100, 22, 10000, **1.9**		58

4. Additional Supplementary Figures

4.1 Energy consuming reaction networks

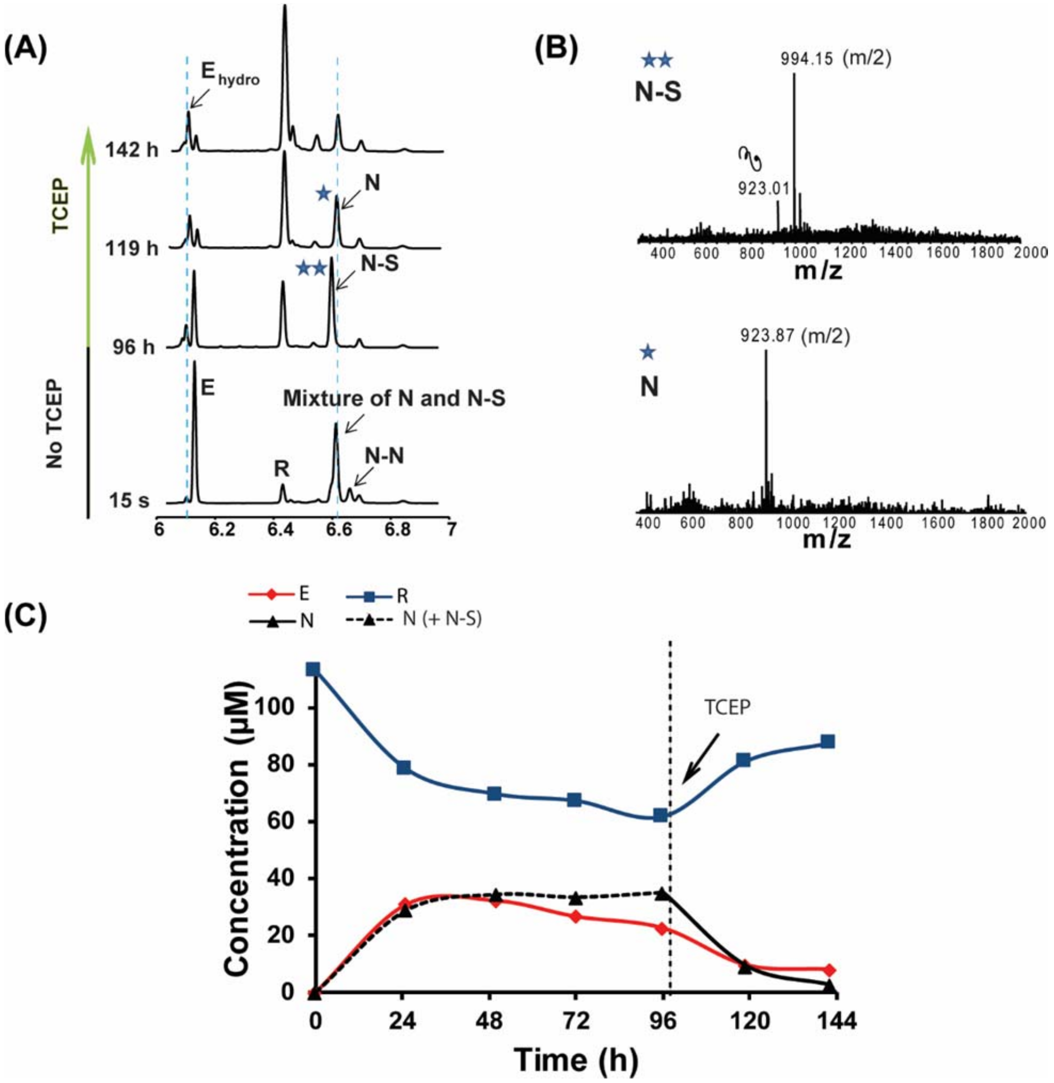

Supplementary Figure 5. (A) UPLC profile over time for the replication reaction (100±10 µM of total peptides) containing ***E***, ***N*** and ***R*** in presence of 10 mM thiol ***S***, at temperature = 35°C. Note the appearance of oxidized species, such as *N-S* and *N-N* in absence of the fuel TCEP, which can turn into the active species ***N*** after the addition of fuel. (B) Mass spectrometry of the 6.65 min peaks from (A) corresponding to the *N-S* mass for the mixture before fuel addition and for the regenerated active ***N*** after the fuel addition. (C) Kinetic profile of the replication reaction initiated with ***R*** only (110 µM). The panel displays the concentrations of ***E***, ***N***, and ***R*** as a function of time. After reaching a resting state (kinetic trap), the reaction network was refueled with TCEP (at 96h) and consequently operated to reach the steady state.

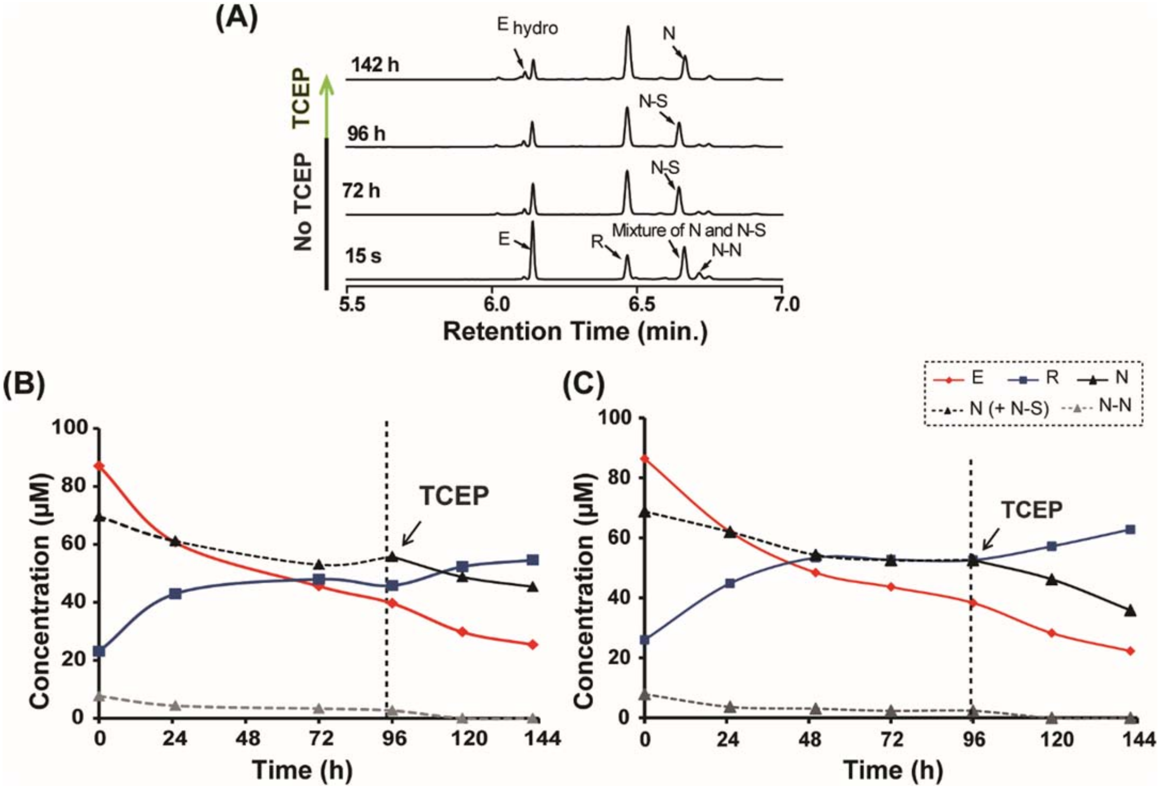

Supplementary Figure 6. (A) UPLC profile of a replication reaction (100±10 μM of total peptides) containing ***E***, ***N*** and ***R*** in presence of 500 μM of thiol, at temperature = 22°C. The replication reaction is initiated without TCEP, and after 96 h TCEP is added to the mixture. (B) and (C) show the corresponding kinetic profiles for a duplicated experiments that were initiated with ~80 μM ***E***, ~80 μM ***N*** and ~20 μM ***R*** displaying the component of ***E***, ***N***, ***R***, *N-S etc*. as a function of time. The replication reactions are trapped with low yield of ***R*** after day 2 in absence of fuel TCEP, but refueling with TCEP allow them to react further towards the SSs.

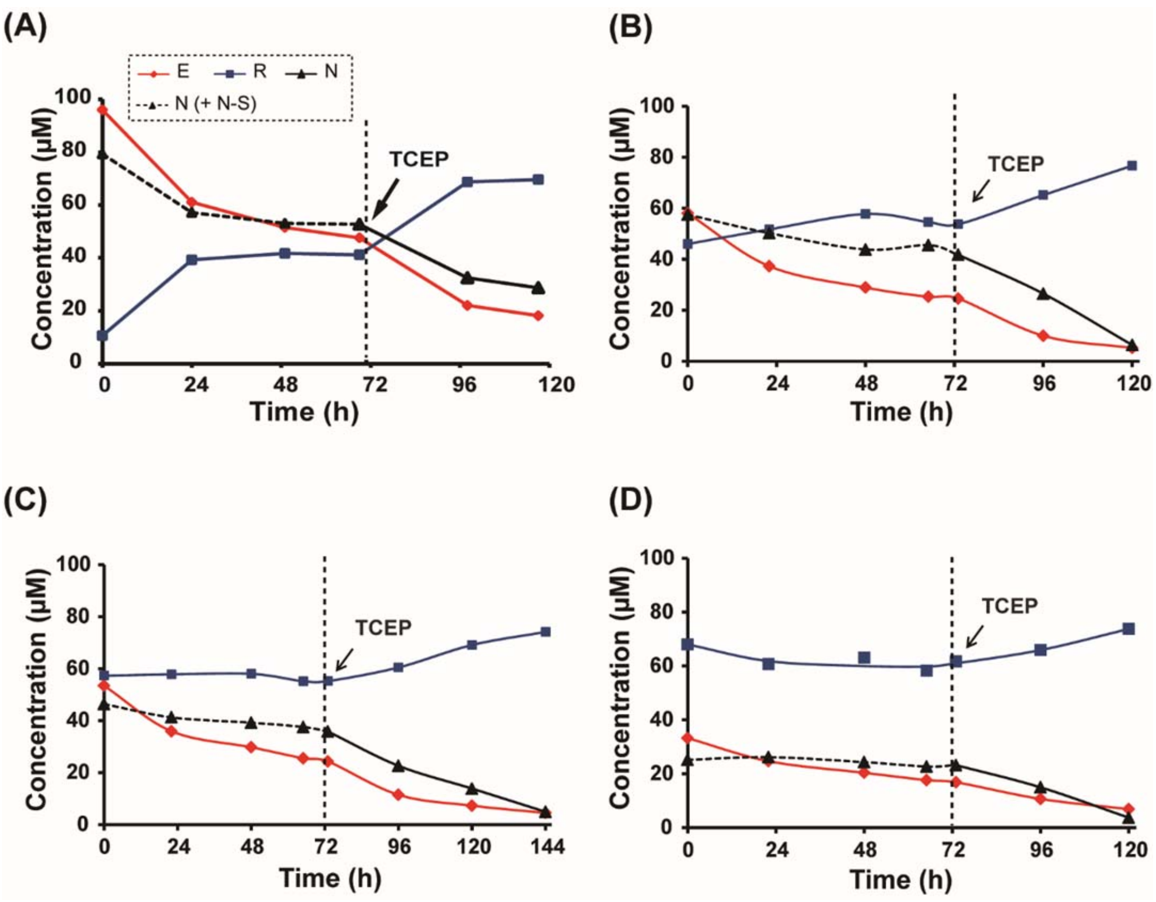

Supplementary Figure 7. Kinetic profile of the replication reactions initiated with (A) ~90 µM ***E***, ~90 µM ***N*** and 10 µM ***R***, (B) 55 µM ***E***, 55 µM ***N*** and 45 µM ***R***, (C) 45 µM ***E***, 45 µM ***N*** and 55 µM ***R*** and (D) 30 µM ***E***, 30 µM ***N*** and 70 µM ***R***. All reactions were performed in presence of 500 µM of thiol, at temperature = 30°C. Note that the replication reactions ceased at early stages in the absence of the fuel TCEP, but were reacting again from the trapped state after the addition of fuel TCEP at 73 h.

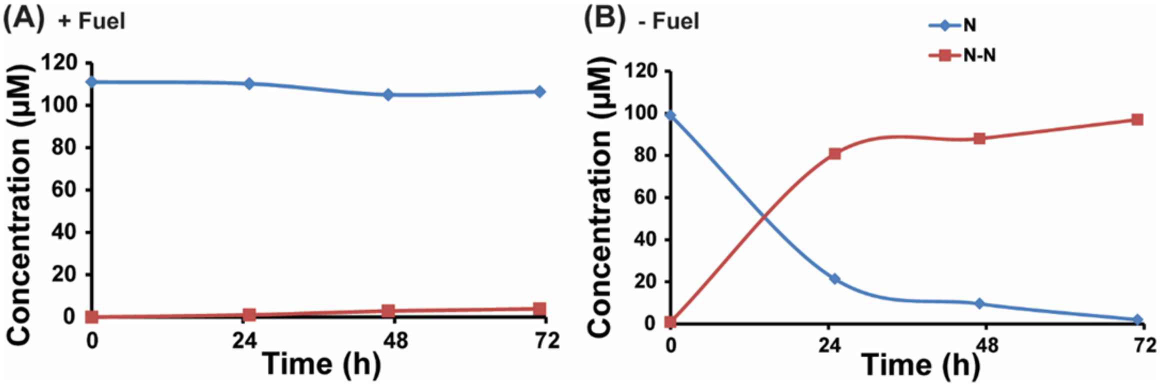

Supplementary Figure 8. A control experiment carried out by reacting the nucleophile ***N*** alone in MOPS buffer. Note that nucleophile remains active when dissolved in the reducing environment (**A**), whereas it was fully converted into its inactive ***N-N*** form within 72 h within the non-reducing environment (**B**).

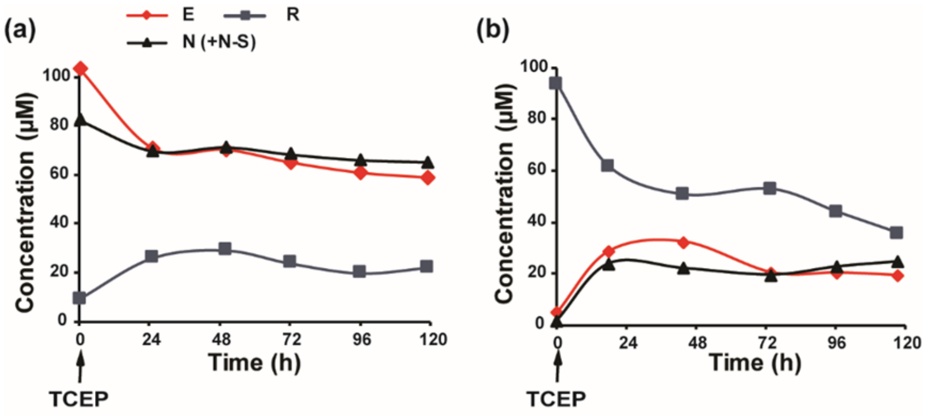

Supplementary Figure 9. Transient profile of the bistable network fuelled with excess TCEP only one time at initiation. The experiments were initiated with 10 µM ***R***, (each 90 ± 10 µM) ***E*** and ***N*** (a), or with 95 µM ***R***, (each 4 ± 2 µM) of ***E*** and ***N*** (b). Reactions were carried out at t = 35 ± 0.2 °C and with [***S***] = 10000 µM.

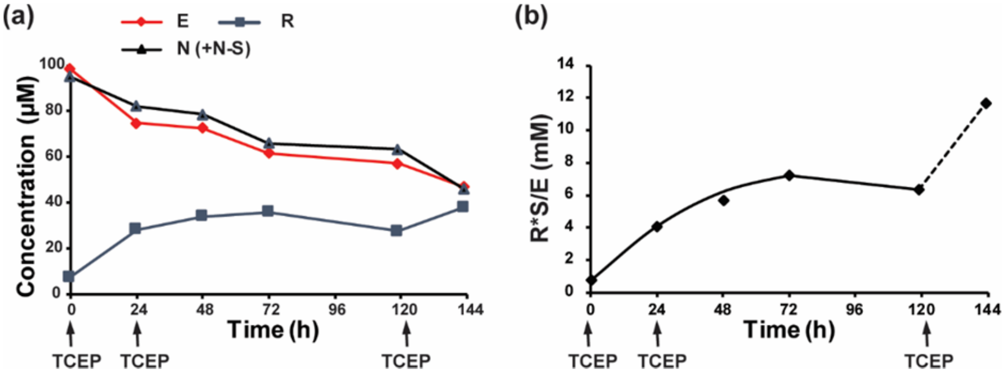

Supplementary Figure 10. Transient profile of the bistable network fuelled three times with excess TCEP, at initiation, after 24h and after 120h. (a) ***E***, ***N*** and ***R*** concentrations as a function of time for a reaction intitated with 10 µM ***R*** and (each 98 ± 2 µM) of ***E*** and ***N***. (b) R*S/E as a function of time calculated from the data in (a). The reaction was carried out at t = 35 ± 0.2 °C and with [***S***] = 10000 µM. 5 mM of TCEP was supplied at each step.

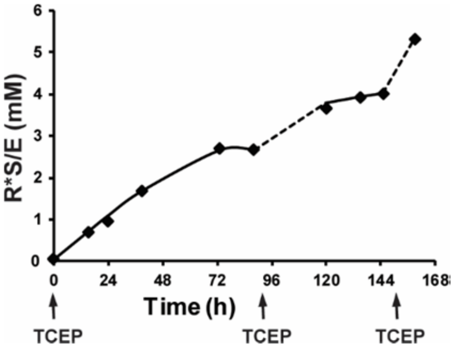

Supplementary Figure 11. R*S/E as function of time for a reaction initiated with 10 µM of ***R*** and (each 90 ± 5 µM) of ***E*** and ***N*** and fueled three times with small amounts of TCEP (2 mM). The reaction was carried out at t = 30°C and [***S***] = 500 µM.

4.2 Computed phase diagrams: The following contour plots were produced using the procedure described above in Sec. 2.3 and the parameters defined in Table s4. The axes of each plot define a parameter space; over this space all stable steady state solutions were located and computed. Each plot contains a bistable region, shown in colour, where two stable steady state solutions were found. The colours for this region correspond, according to the accompanying colour table, to the ΔK_{app} values at each point. Outside the bistable region, where only one stable steady state solution was found, the K_{app} values are shown in grey scale, corresponding to the accompanying grey table.

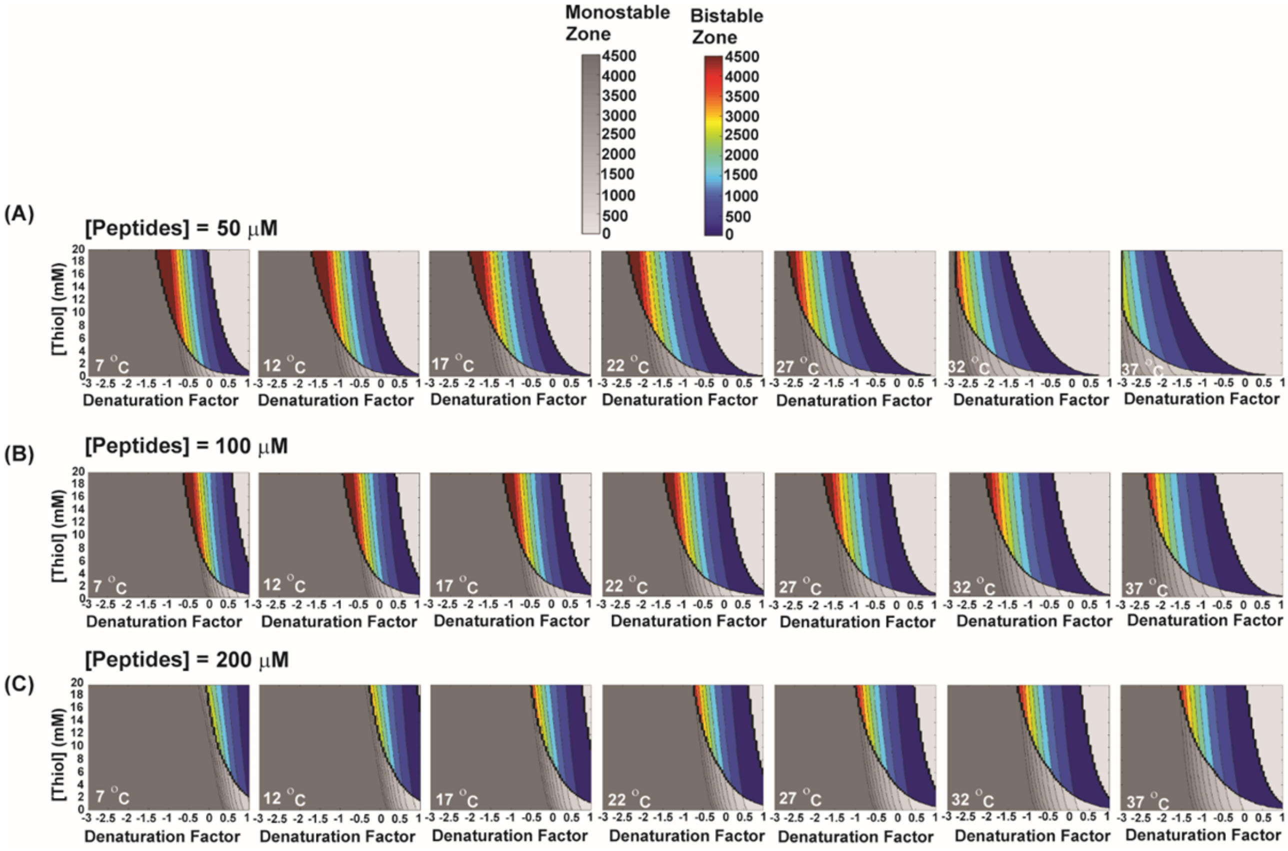

Supplementary Figure 12. **(A), (B)** and **(C)** display the phase diagrams for the degree of SS separation, computed over the parameters space, enabling location of the bistable zones.

4.3 UPLC Chromatograms of the network reactions at initiation and steady states

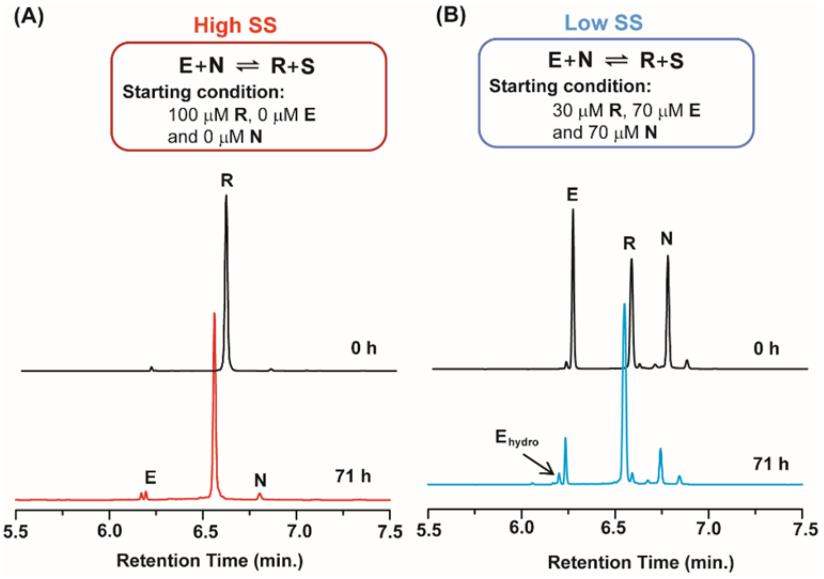

Supplementary Figure 13. UPLC chromatograms obtained for reactions that were carried out with total [peptide] (E+R) = 100 μM, temperature = 22 ± 0.2 °C, [thiol] = 500 μM and without any denaturant.

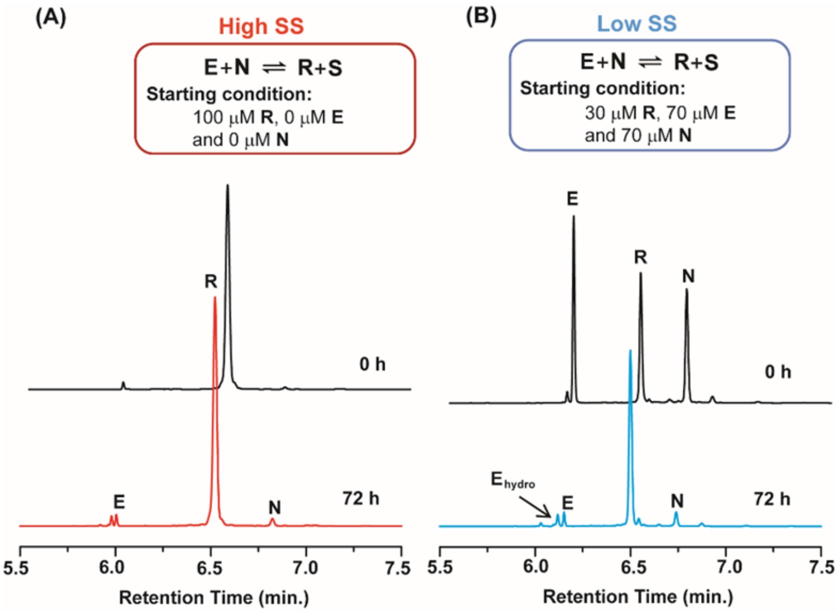

Supplementary Figure 14. UPLC chromatograms obtained for reactions that were carried out with [peptides] = 100 μM, t = 30 ± 0.2 °C, [thiol] = 500 μM and without any denaturant.

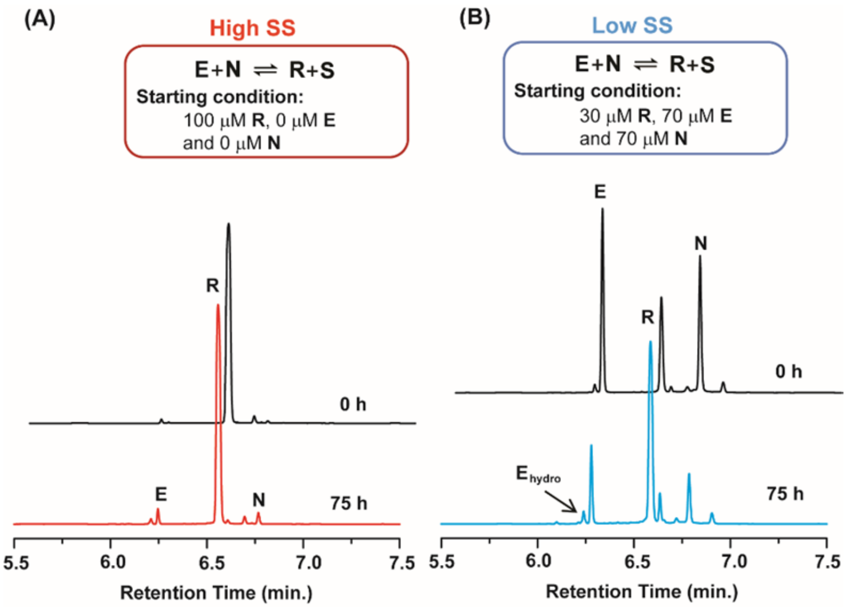

Supplementary Figure 15. UPLC chromatograms obtained for reactions that were carried out with total [peptides] = 100 μM, at t = 22 ± 0.2 °C, [thiol] = 500 μM and [GnHCl] = 0.5 M.

4.4 Networks responses to changes in various control parameters

(i) 'Native' conditions

Case 1

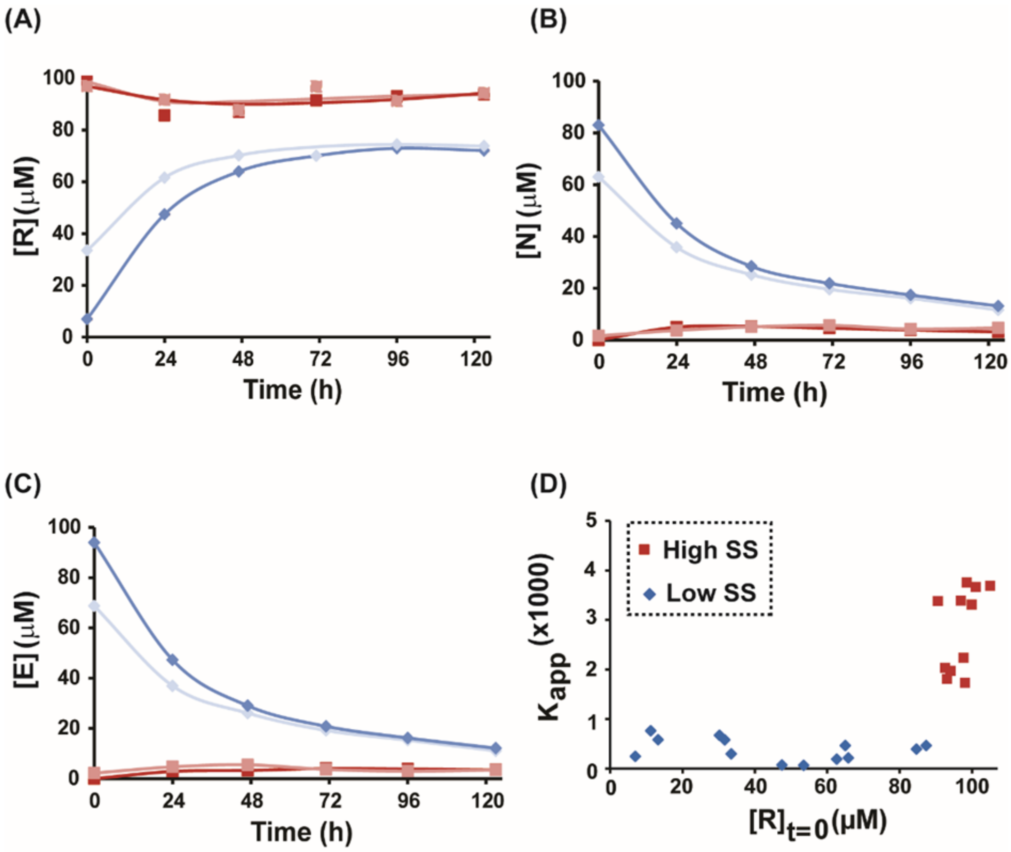

Supplementary Figure 16. (A), (B) and (C) are the ***R***, ***N*** and ***E*** concentrations as a function of time for representative reactions leading to low (dark & light *blue* traces) or high (dark & light *red* traces) SS concentration distributions. Reactions were carried out with total [peptides] = 100 μM concentration, at t = 22 ± 0.2 °C and [thiol] = 500 μM. **(D)** represents the K_{app} values as a function of the initial concentration of ***R***, highlighting low (*blue*) and high (*red*) SS distributions.

(ii) Varying reaction temperatures

Case 2

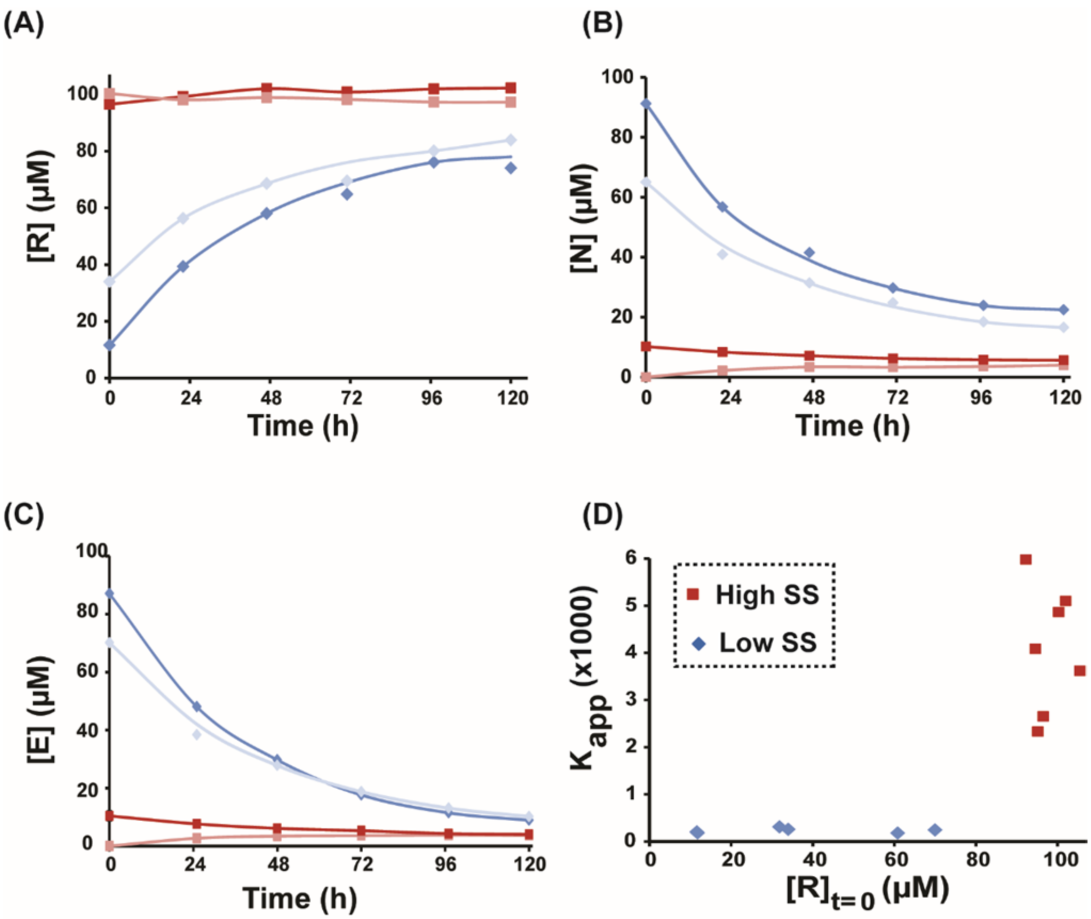

Supplementary Figure 17. (A), (B) and **(C)** are the ***R***, ***N*** and ***E*** concentrations as a function of time for representative reactions leading to low (dark & light *blue* traces) or high (dark & light *red* traces) SS concentration distributions. Reactions were carried out with total [peptides] = 100 μM, at t = 12 ± 0.2 °C and [thiol] = 500 μM. **(D)** represents the K_{app} values as a function of the initial concentration of ***R***, highlighting low (*blue*) and high (*red*) SS distributions.

Case 3

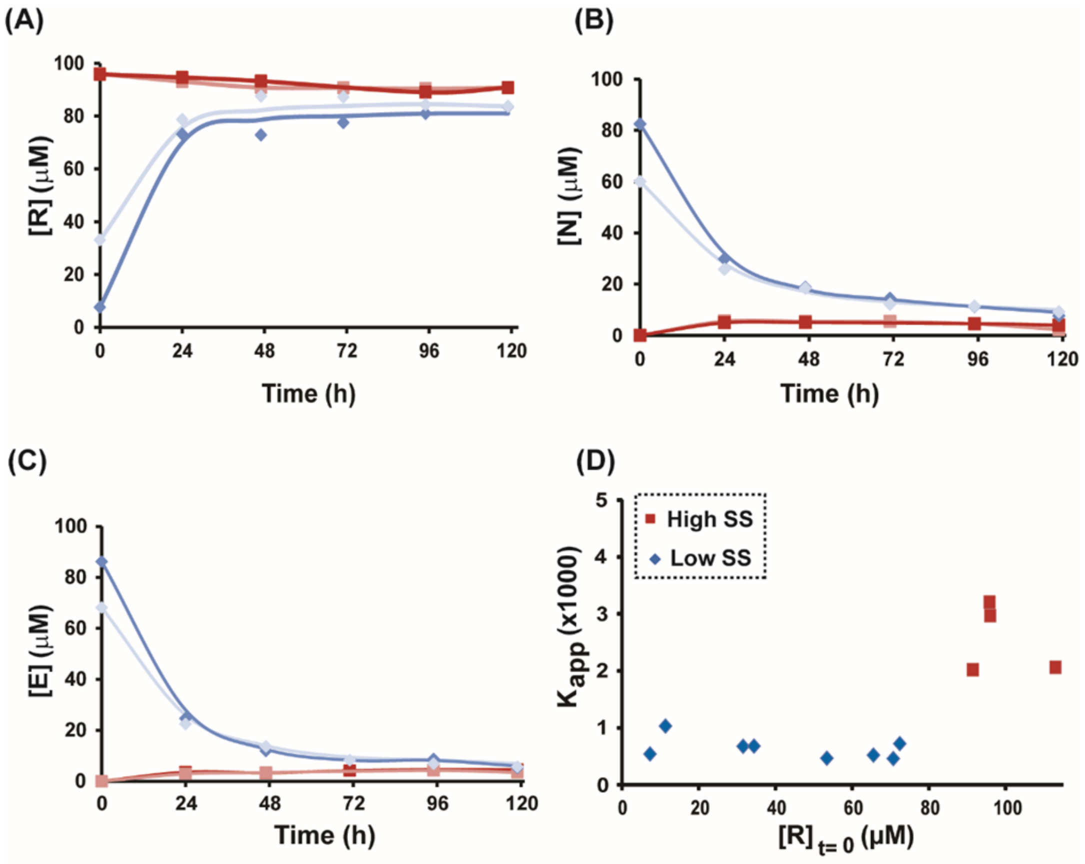

Supplementary Figure 18. (A), (B) and **(C)** are the ***R***, ***N*** and ***E*** concentrations as a function of time for representative reactions leading to low (dark & light *blue* traces) or high (dark & light *red* traces) SS concentration distributions. Reactions were carried out with total [peptides] = 100 µM, at t = 30 ± 0.2 °C and [thiol] = 500 µM. **(D)** represents the K_{app} values as a function of the initial concentration of ***R***, highlighting low (*blue*) and high (*red*) SS distributions.

Case 4

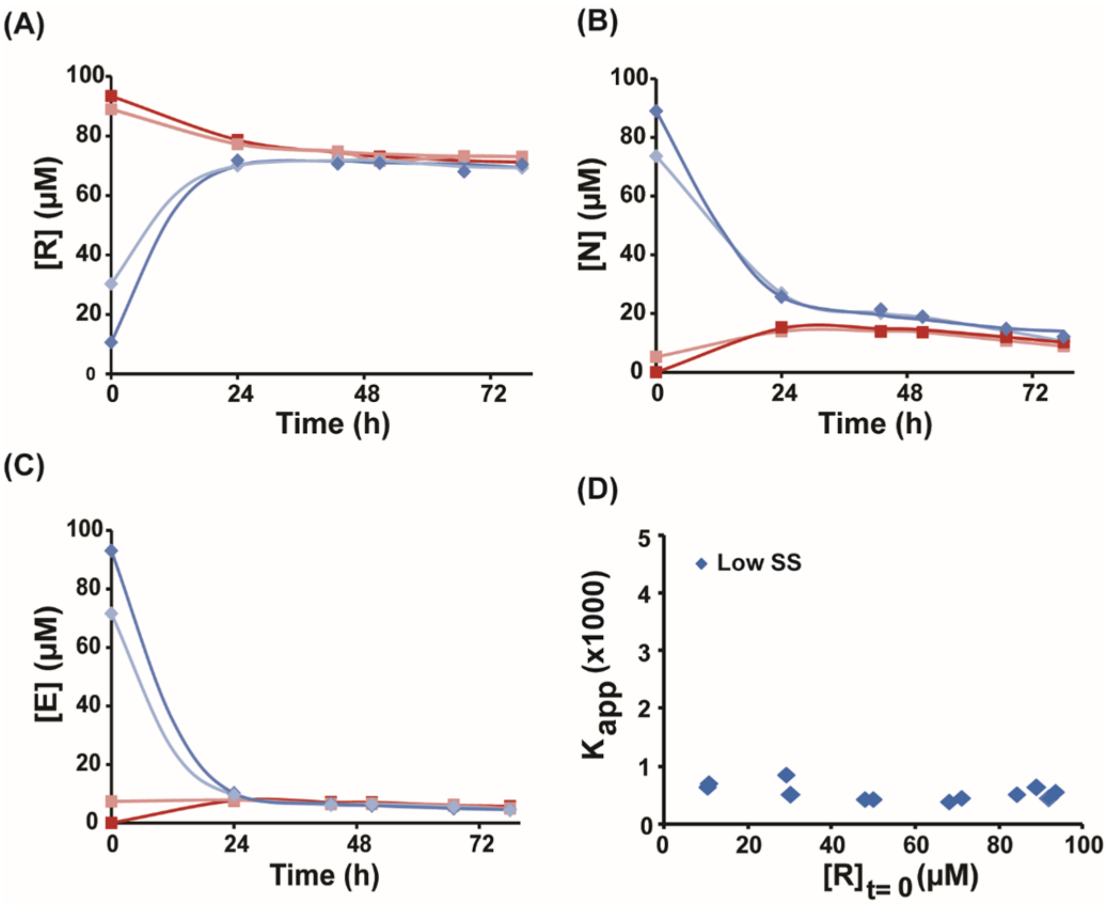

Supplementary Figure 19. (A), (B) and (C) are the ***R***, ***N*** and ***E*** concentrations as a function of time. Reactions were carried out with total [peptides] = 100 μM, at t = 35 ± 0.2 °C and [thiol] = 500 μM. **(D)** represents the K_{app} values as a function of the initial concentration of ***R***, highlighting low SS distributions, signifying the monostable network.

Case 5

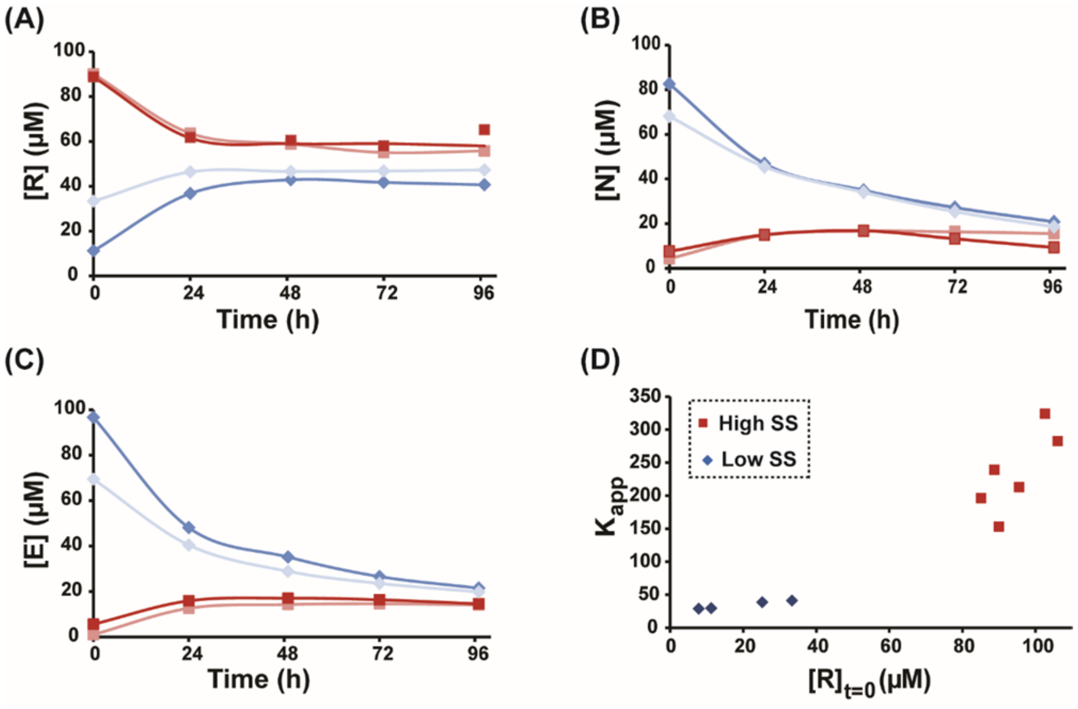

Supplementary Figure 20. (A), (B) and (C) are the ***R***, ***N*** and ***E*** concentrations as a function of time for representative reactions leading to low (dark & light *blue* traces) or high (dark & light *red* traces) SS concentration distributions. The reactions were carried out with total [peptides] = 100 μM, at t = 37 ± 0.2 °C and [thiol] = 500 μM. **(D)** represents the K_{app} values as a function of the initial concentration of ***R***, highlighting low (*blue*) and high (*red*) SS distributions. Note that in this case the electrophile ***E*** was hydrolyzed faster than in lower temperature (maximum hydrolysis 20%).

(iii) Varying the total peptide concentrations

<u>Case 6</u>

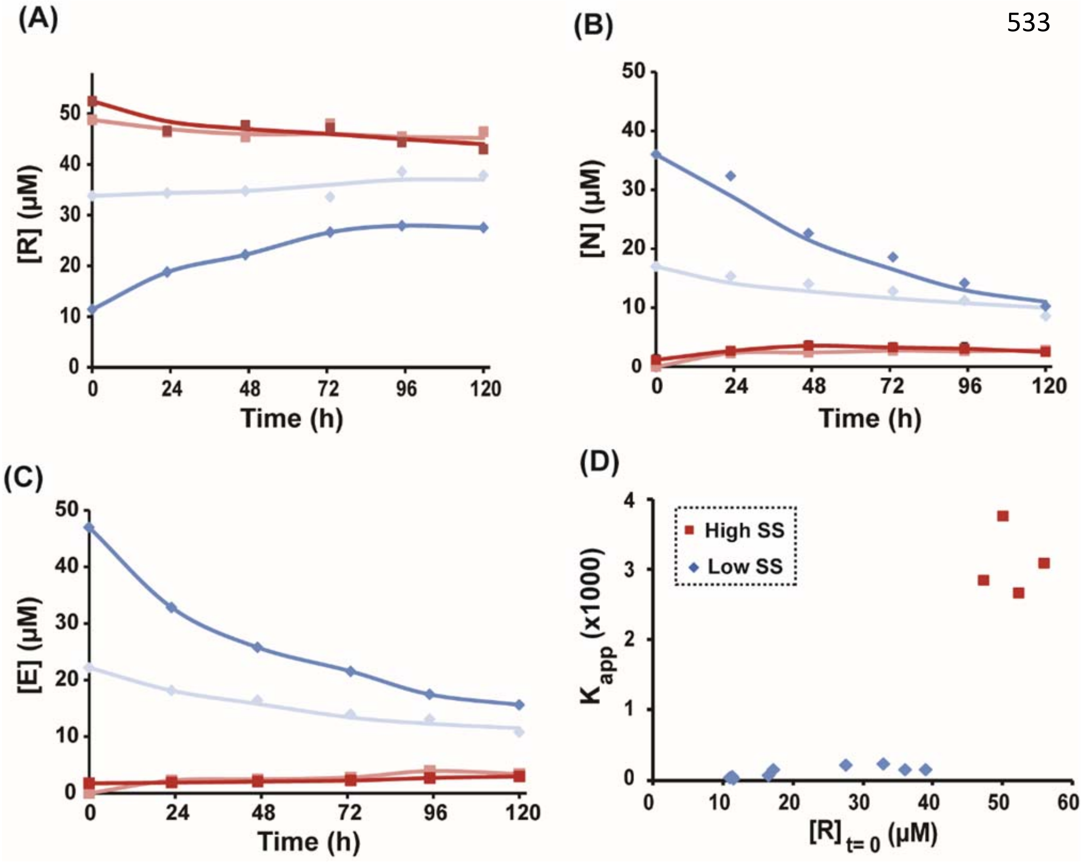

Supplementary Figure 21. **(A), (B)** and **(C)** are the ***R***, ***N*** and ***E*** concentrations as a function of time for representative reactions leading to low (dark & light *blue* traces) or high (dark & light *red* traces) SS concentration distributions. Reactions were carried out with total [peptides] (E+R) = 50 µM, at t = 22 ± 0.2 °C and [thiol] = 500 µM. **(D)** represents the K_{app} values as a function of the initial concentration of ***R***, highlighting low (*blue*) and high (*red*) SS distributions.

Case 7

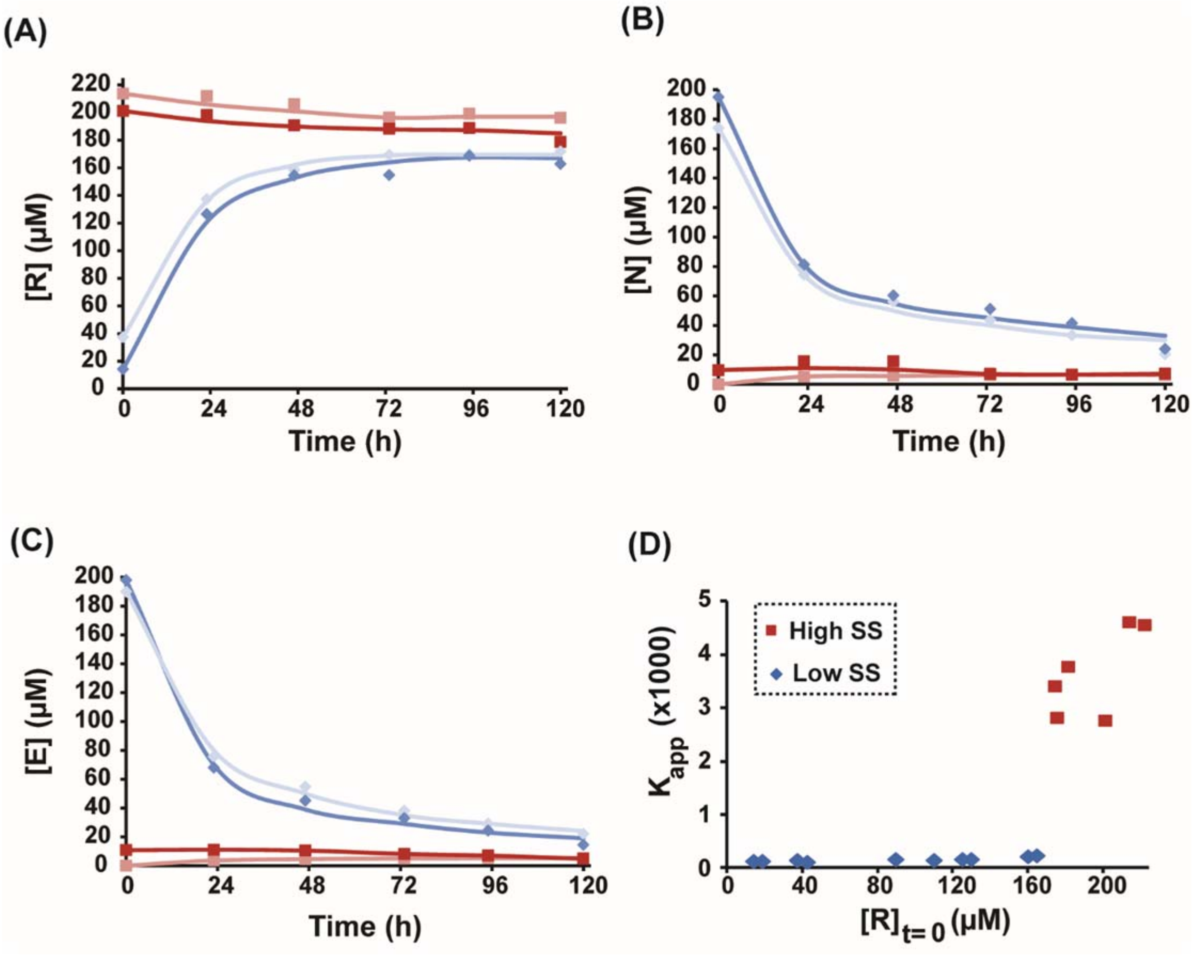

Supplementary Figure 22. **(A), (B**) and **(C)** are the ***R***, ***N*** and ***E*** concentrations as a function of time for representative reactions leading to low (dark & light *blue* traces) or high (dark & light *red* traces) SS concentration distributions. Reactions were carried out with total [peptides] = 200 µM, at t = 22 ± 0.2 °C and [thiol] = 500 µM. **(D)** represents the K_{app} values as a function of the initial concentration of ***R***, highlighting low (*blue*) and high (*red*) SS distributions.

(iv) Varying thiol concentrations

Case 8

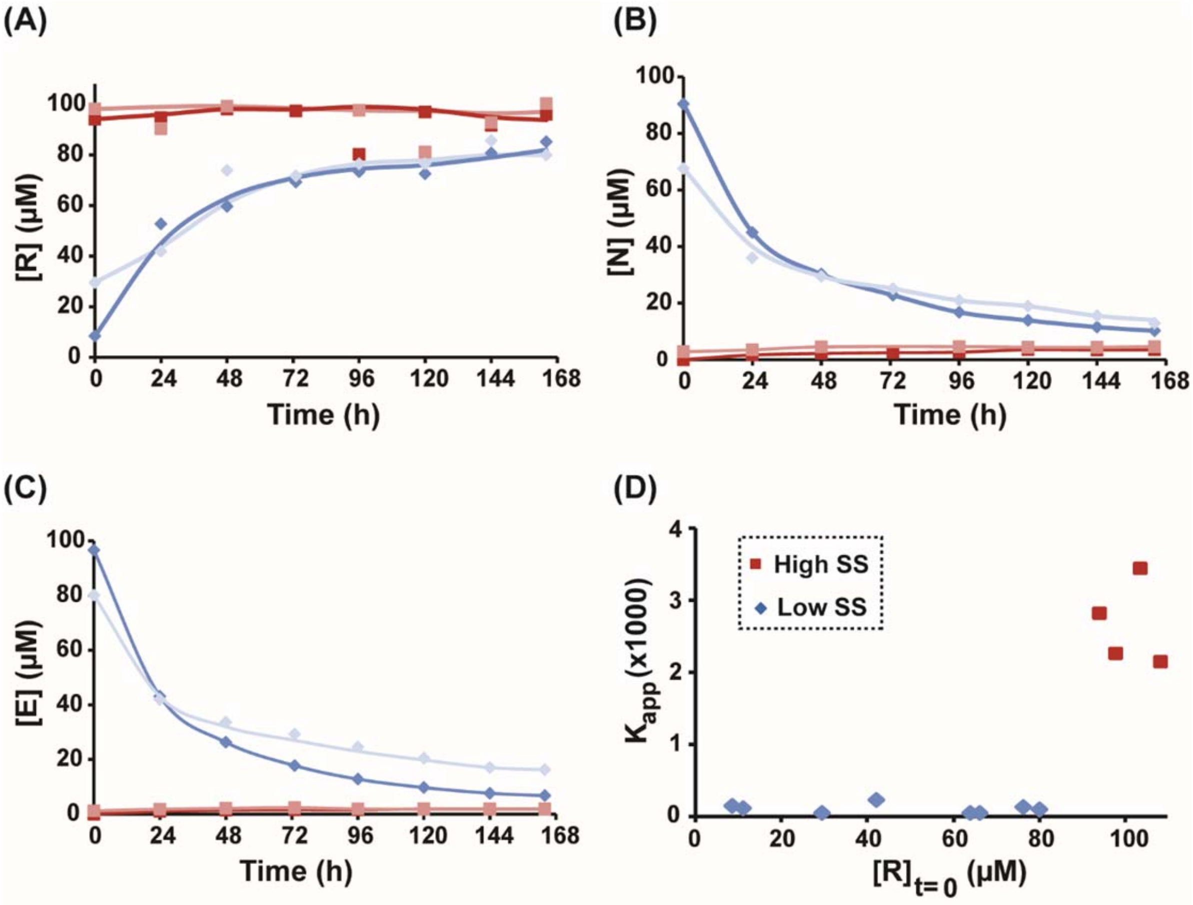

Supplementary Figure 23. (A), (B) and **(C)** are the ***R***, ***N*** and ***E*** concentrations as a function of time for representative reactions leading to low (dark & light *blue* traces) or high (dark & light red traces) SS concentration distributions. Reactions were carried out with total [peptides] = 100 μM, at t = 22 ± 0.2 °C and [thiol] = 100 μM. **(D)** represents the K_{app} values as a function of the initial concentration of ***R***, highlighting low (*blue*) and high (*red*) SS distributions.

Case 9

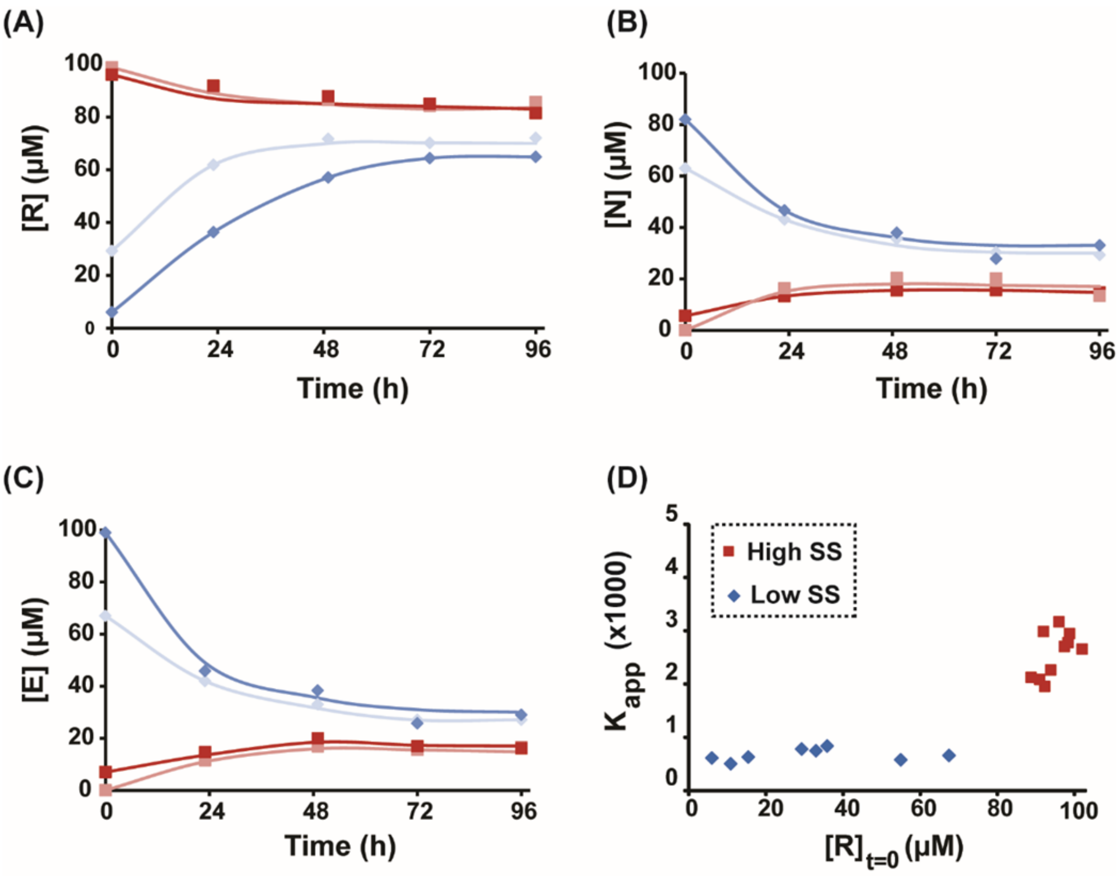

Supplementary Figure 24. (A), (B) and (C) are the ***R***, ***N*** and ***E*** concentrations as a function of time for representative reactions leading to low (dark & light *blue* traces) or high (dark & light *red* traces) SS concentration distributions. Reactions were carried out with total [peptides] = 100 µM, at t = 22 ± 0.2 °C and [thiol] = 10000 µM. **(D)** represents the K_{app} values as a function of the initial concentration of ***R***, highlighting low (*blue*) and high (*red*) SS distributions.

(v) Varying denaturant (GnHCl) concentrations

<u>Case 10</u>

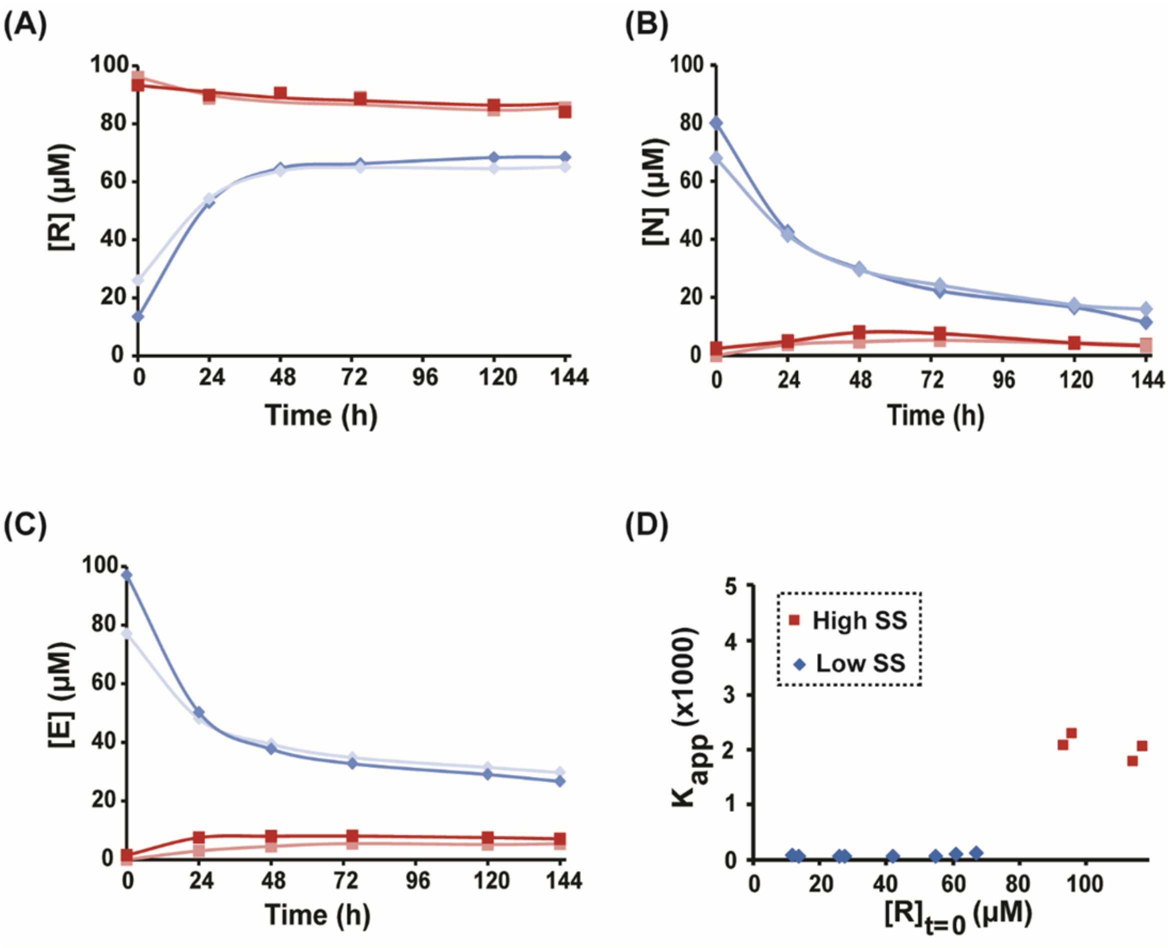

Supplementary Figure 25. (A), (**B**) and **(C)** are the ***R***, ***N*** and ***E*** concentrations as a function of time for representative reactions leading to low (dark & light *blue* traces) or high (dark & light *red* traces) SS concentration distributions. Reactions were carried out with total [peptides] = 100 µM, at t = 22 ± 0.2 °C, [thiol] = 500 µM and [GnHCl] = 0.5 M. **(D)** represents the K_{app} values as a function of the initial concentration of ***R***, highlighting low (*blue*) and high (*red*) SS distributions.

Case 11

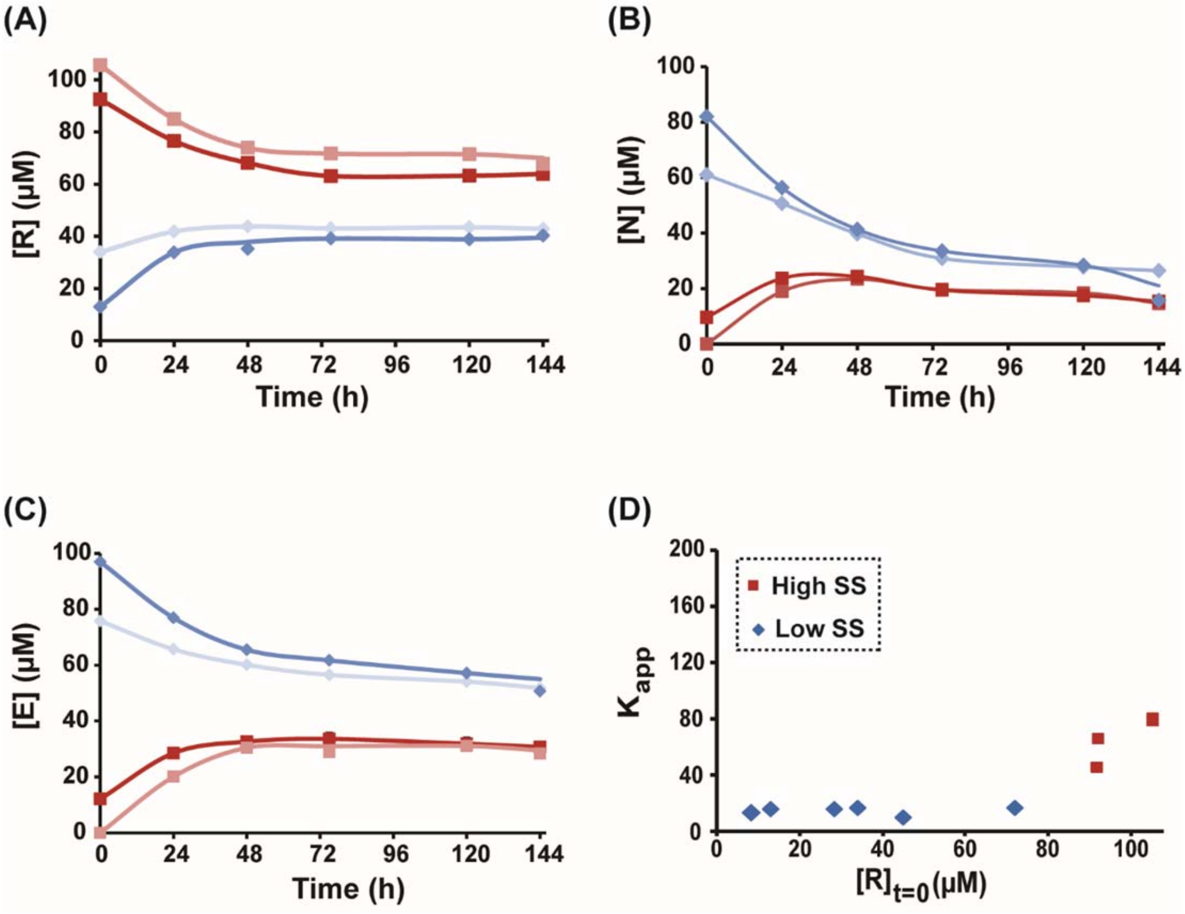

Supplementary Figure 26. (A), (B) and **(C)** are the ***R***, ***N*** and ***E*** concentrations as a function of time for representative reactions leading to low (dark & light *blue* traces) or high (dark & light *red* traces) SS concentration distributions. Reactions were carried out with total [peptides] = 100 µM, at t = 22 ± 0.2 °C, [thiol] = 500 µM and [GnHCl] = 1.5 M. **(D)** represents the K_{app} values as a function of the initial concentration of ***R***, highlighting low (*blue*) and high (*red*) SS distributions.

Case 12

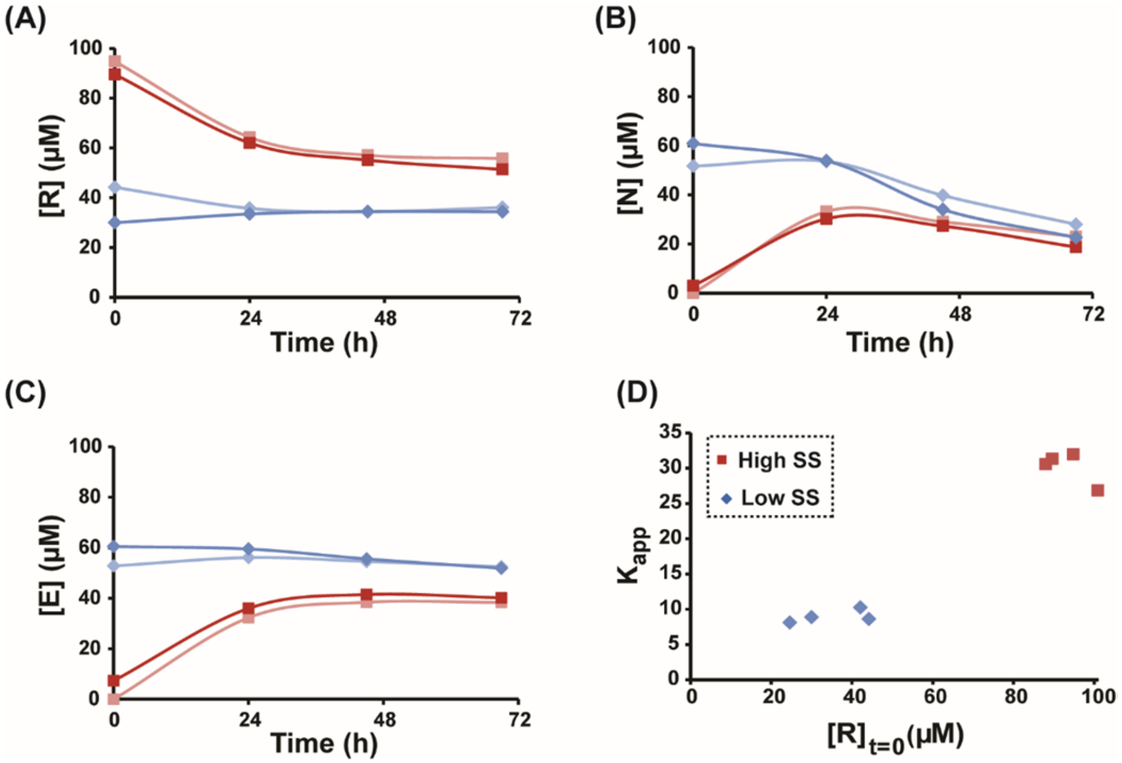

Supplementary Figure 27. (A), (B) and **(C)** are the ***R***, ***N*** and ***E*** concentrations as a function of time for representative reactions leading to low (dark & light *blue* traces) or high (dark & light *red* traces) SS concentration distributions. Reactions were carried out with total [peptides] = 100 μM, at t = 22 ± 0.2 °C, [thiol] = 500 μM and [GnHCl] = 1.8 M. **(D)** represents the K_{app} values as a function of the initial concentration of ***R***.

Case 14

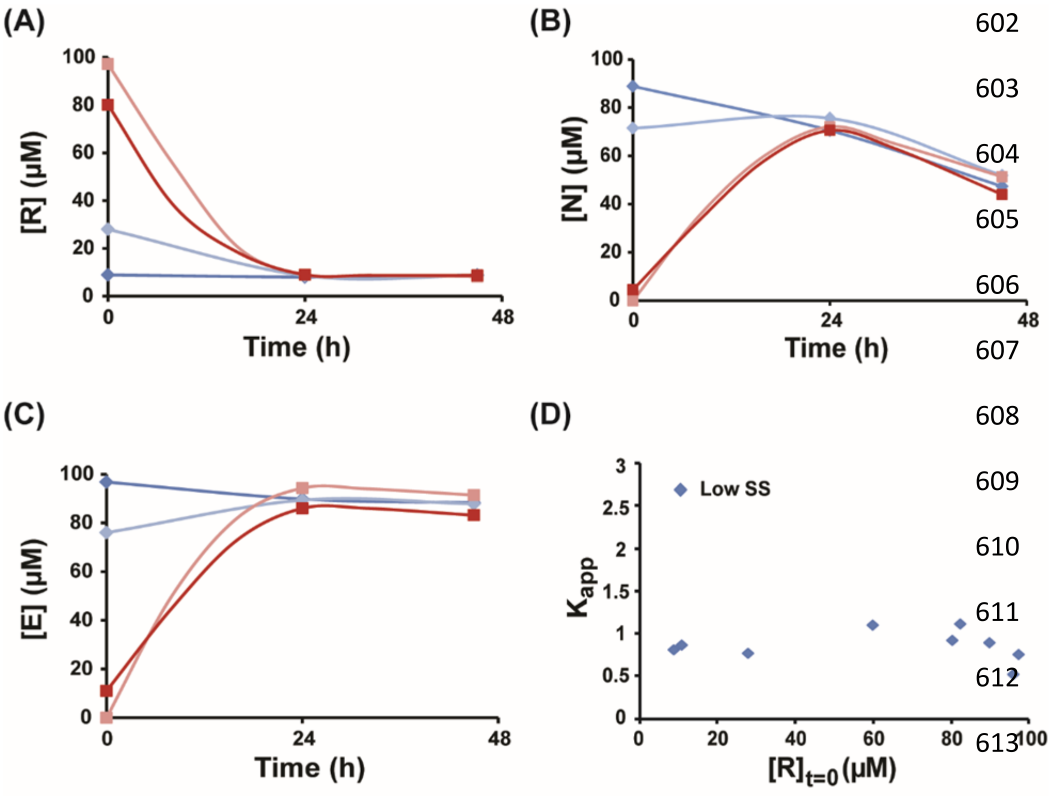

Supplementary Figure 28. (A), (B) and (C) are the ***R***, ***N*** and ***E*** concentrations as a function of time. Reactions were carried out with total [peptides] = 100 µM, at t = 22 ± 0.2 °C, [thiol] = 500 µM and [GnHCl] = 3 M. **(D)** represents the K_{app} values as a function of the initial concentration of ***R***, signifying the low (*blue*) SS distributions of a monostable network.

(vi) Varying replicator peptide sequence

<u>Case 13</u>

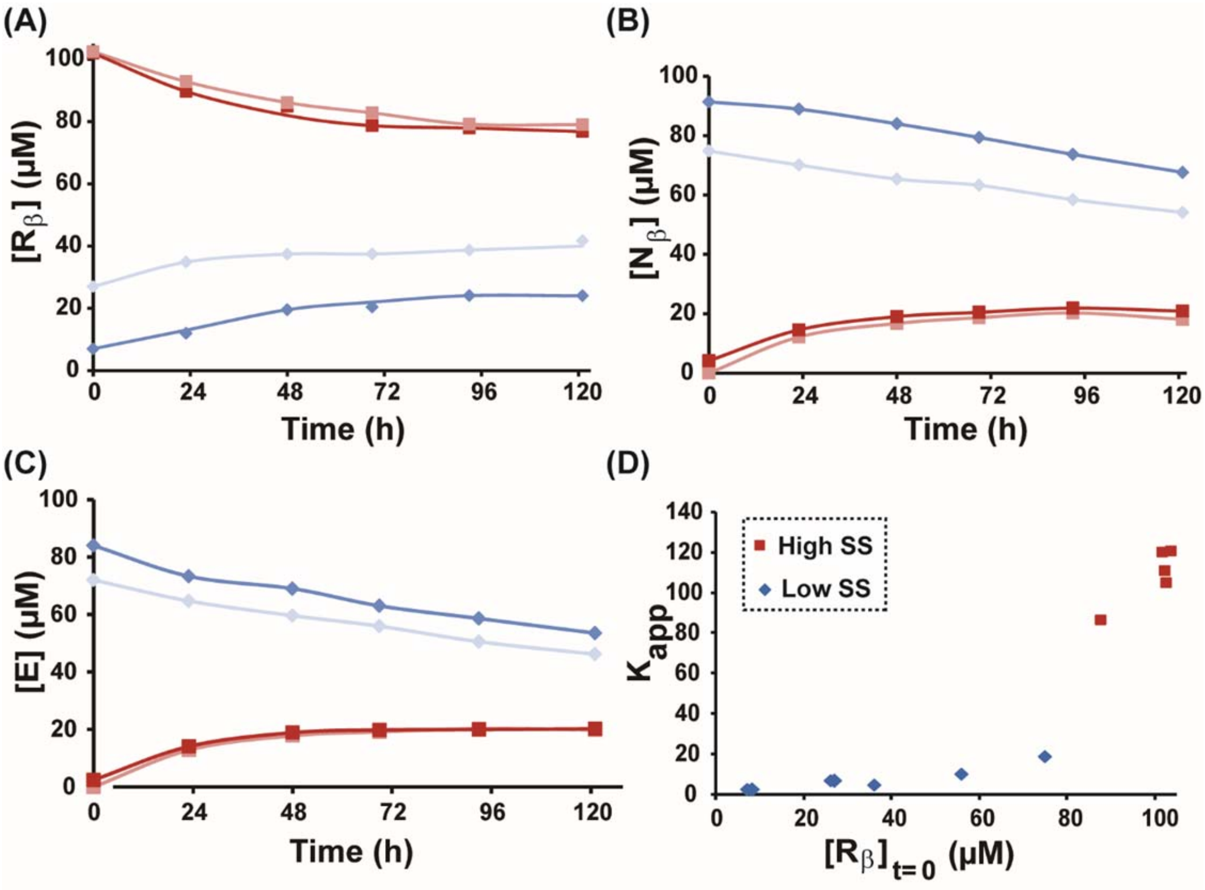

Supplementary Figure 29. **(A), (B)** and **(C)** are the $\boldsymbol{R_\beta}$, $\boldsymbol{N_\beta}$ and ***E***, concentrations as a function of time for representative reactions leading to low (dark & light *blue* traces) or high (dark & light *red* traces) SS concentration distributions. Reactions were carried out with total [peptides] ($E+R_\beta$) = 100 μM, at t = 22 ± 0.2 °C and [thiol] = 500 μM. Unlike the other above-presented cases, here the replicator and nucleophile are $\boldsymbol{R_\beta}$ and $\boldsymbol{N_\beta}$ rather than replicator ***R*** and nucleophile ***N***. Peptide sequences: $\boldsymbol{N_\beta}$ = H-ZLEXEVARLKKLVGE-CONH$_2$**,** $\boldsymbol{R_\beta}$ =Ar-RVARLEKKVSALEKKVAZLEXEVA-RLKKLVGE-CONH$_2$**,** Ar = 4-acetamidobenzoate, Z=-SCH$_2$CH$_2$CO, X = Lys-Ar, SR = 2-mercapto-ethane sulfonate**.** **(D)** represents the K_{app} values as a function of the initial concentration of $\boldsymbol{R_\beta}$, highlighting low (*blue*) and high (*red*) SS distributions.

(vii) Addition of a Kosmotropic salt

<u>Case 15</u>

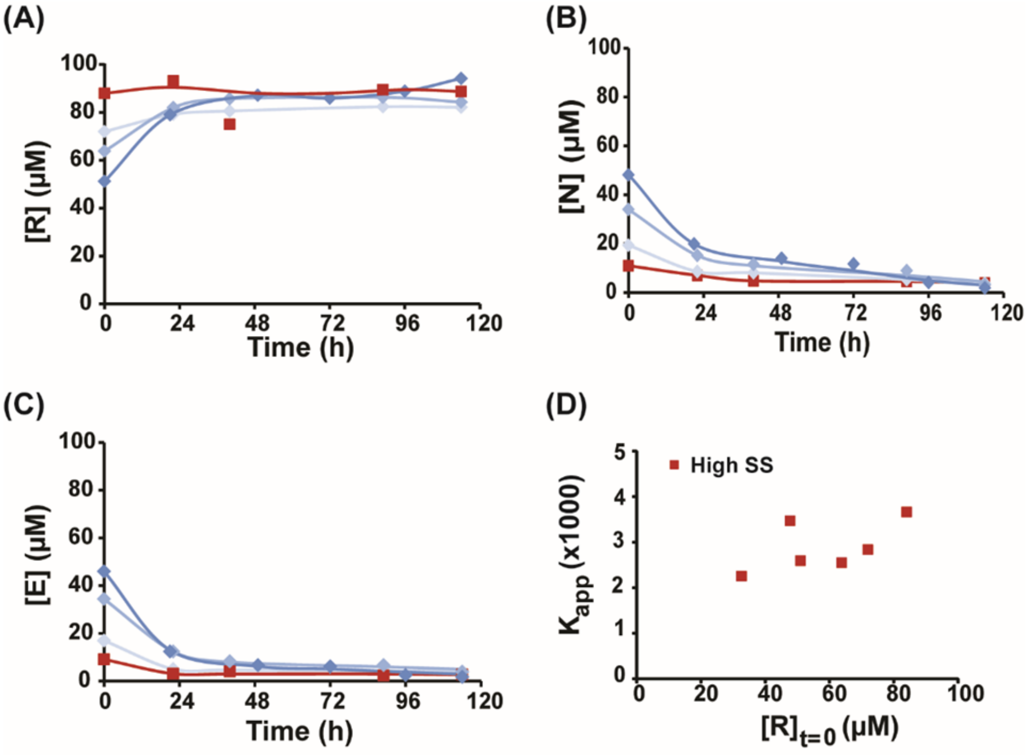

Supplementary Figure 30. (A), (B) and **(C)** are the ***R***, ***N*** and ***E*** concentrations as a function of time. Reactions were carried out with total [peptides] = 100 µM, at t = 22 ± 0.2 °C and [thiol] = 500 µM. In this case, the conditions were varied from the native conditions (case 1) by the addition of 0.5 M Kosmotropic salt Na_2SO_4. **(D)** represents the K_{app} values as a function of the initial concentration of ***R***, signifying high (*red*) SS distributions of the monostable network.

(viii) Varying thiol concentration and temperature simultaneously

<u>Case 16</u>

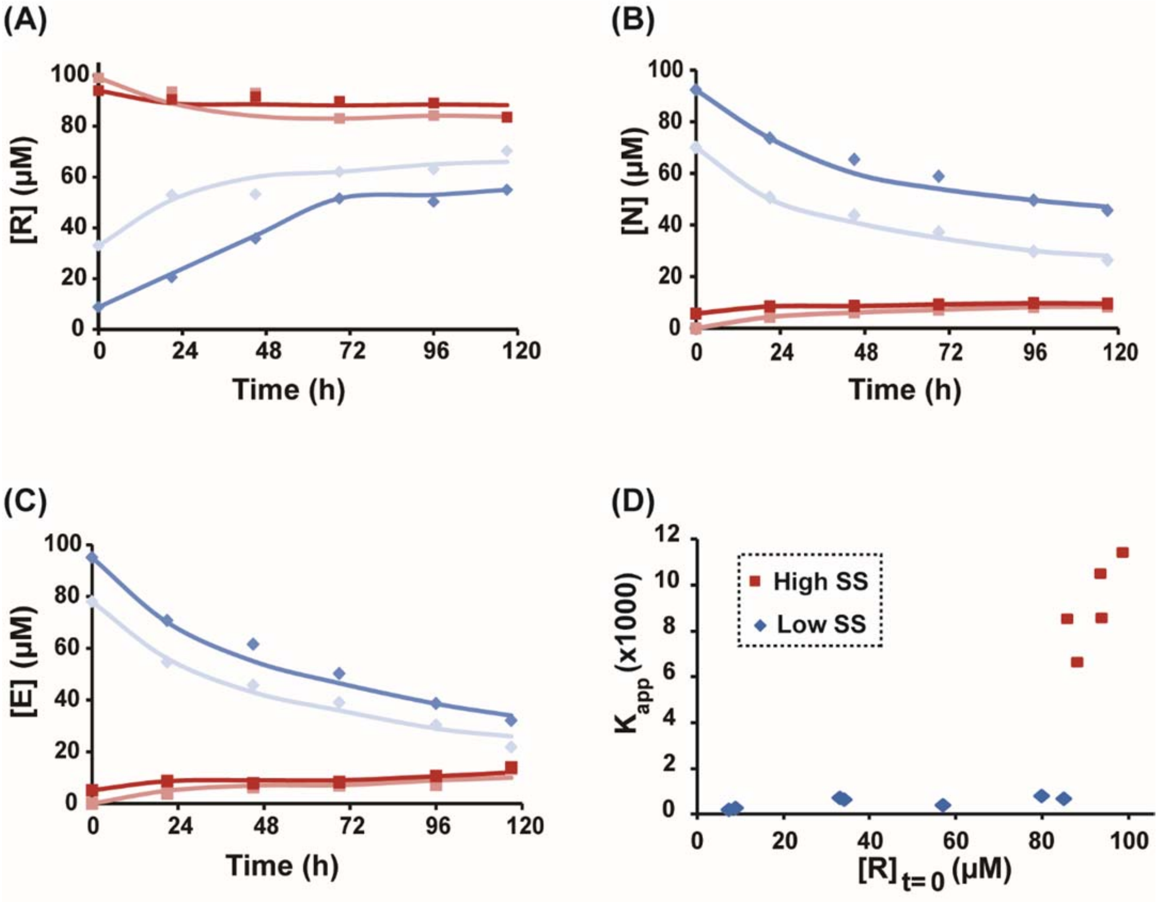

Supplementary Figure 31. (A), (B) and **(C)** are the ***R***, ***N*** and ***E*** concentrations as a function of time for representative reactions leading to low (dark & light *blue* traces) or high (dark & light *red* traces) SS concentration distributions. Reactions were carried out with total [peptides] = 100 μM, at t = 12 ± 0.2 °C and [thiol] = 10000 μM. **(D)** represents the K_{app} values as a function of the initial concentration of ***R***, highlighting low (*blue*) and high (*red*) SS distributions.

Case 17

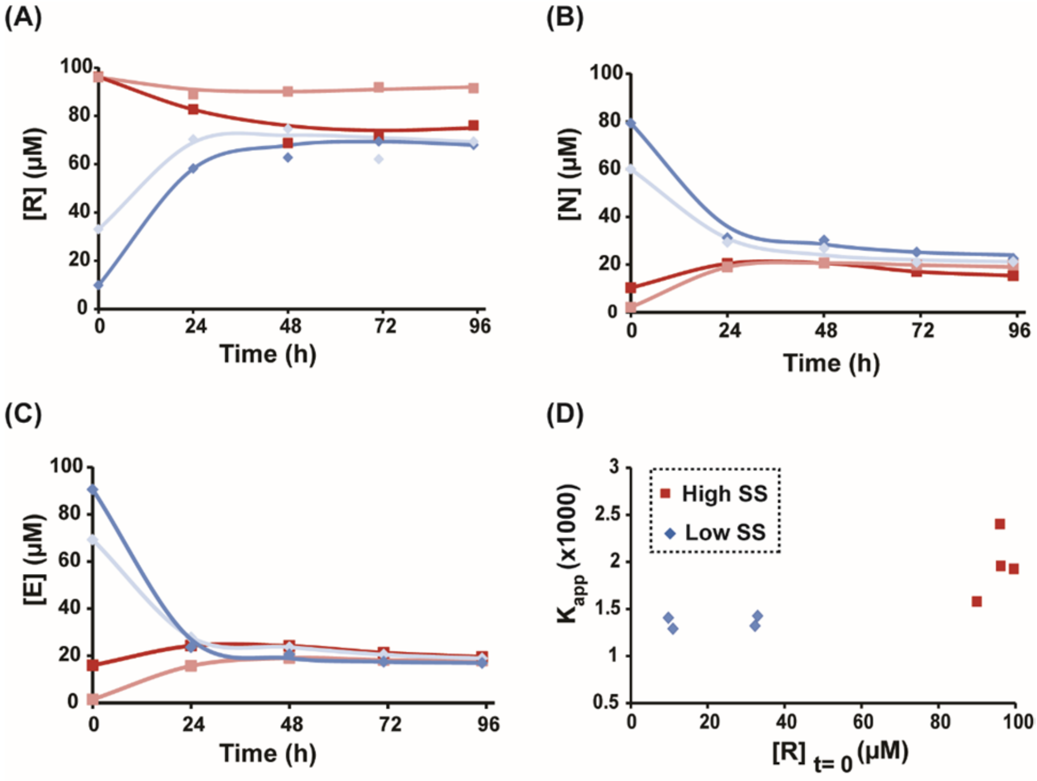

Supplementary Figure 32. (A), (B) and **(C)** are the ***R***, ***N*** and ***E*** concentrations as a function of time for representative reactions leading to low (dark & light *blue* traces) or high (dark & light *red* traces) SS concentration distributions. Reactions were carried out with total [peptides] = 100 μM, at t = 30 ± 0.2 °C and [thiol] = 10000 μM. **(D)** represents the K_{app} values as a function of the initial concentration of ***R***, highlighting low (*blue*) and high (*red*) SS distributions.

Case 18

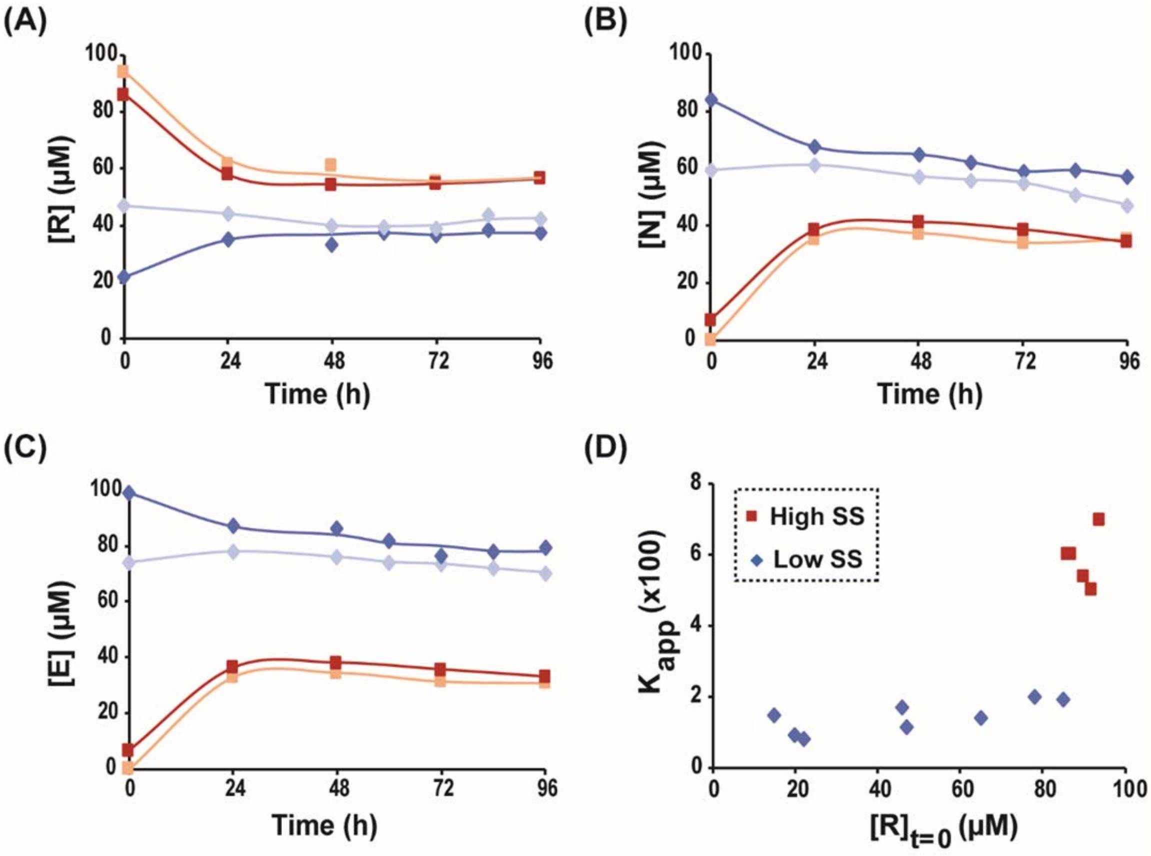

Supplementary Figure 33. (A), (B) and **(C)** are the ***R***, ***N*** and ***E*** concentrations as a function of time for representative reactions leading to low (dark & light *blue* traces) or high (dark & light *red* traces) SS concentration distributions. Reactions were carried out with total [peptides] = 100 μM, at t = 35 ± 0.2 °C and [thiol] = 10000 μM. **(D)** represents the K_{app} values as a function of the initial concentration of ***R***, highlighting low (*blue*) and high (*red*) SS distributions.

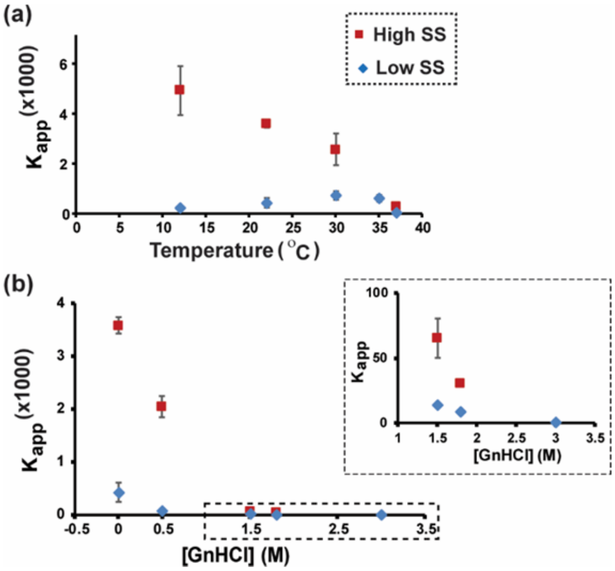

Supplementary Figure 34. K_{app} values as a function of the temperature (a) and GnHCl concentration (b). Reactions in (a) were carried out at various temperatures with total [peptide] = 100 μM, and [thiol] = 500 μM, without GnHCl; reactions in (b) carried out with different GnHCl concentrations, total [peptides] = 100 μM, at t = 22 ± 0.2 °C and [thiol] = 500 μM.

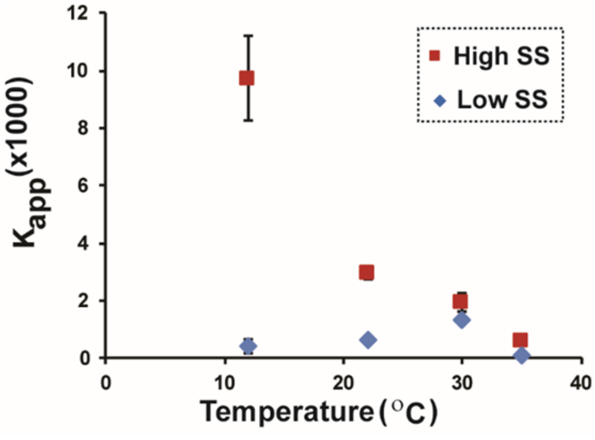

Supplementary Figure 35. K_{app} values as a function of the temperature. Reactions were carried out at various temperatures with total [peptides] = 100 μM, and [thiol] = 10000 μM, without GnHCl.

4.5 Circular dichroism (CD) and helical propensity

(a)

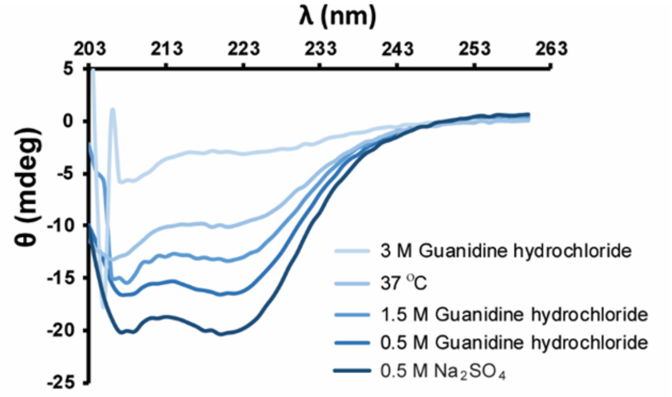

(b)

Type of replicator	Experimental case	Experimental Condition	% Helicity
R	14	3 M Guanidine hydrochloride	19.2
R	5	37 °C	44.9
R_β [6]	13	22 °C	51
R [int]	12	1.8 M Guanidine hydrochloride	54.9
R	11	1.5 M Guanidine hydrochloride	57
R	10	0.5 M Guanidine hydrochloride	68.5
R [7]	1	22 °C	70.1
R	15	0.5 M Na_2SO_4	82.3

Supplementary Figure 36. (a) CD spectra obtained for 25 µM ***R*** and ***R***$_\beta$ at various conditions. The experiments were performed in 5 mM MOPS buffer pH 7. (b) A table summarizing the percentage of helical content of ***R*** and ***R***$_\beta$ under each condition. Measurements were carried out on a Jasco-815 CD spectropolarimeter, at 22 °C (case #5 only at 37 °C), by using a quartz cell with 1.0 mm path length. CD spectra were obtained as the average of three scans and collecting data at 1 nm intervals from 260 to 200 nm. The helical content of each peptide was calculated using CDNN program (provided with the Jasco spectropolarimeter). For case #12 the helicity value was intrapulated from the data obtained in different concentrations of GnHCl. Case #13 is shown in Figure 5 of the main manuscript after cases #11 and #12, as it shows larger extent of bistability.

5. Supplementary references:

1. Wagner, N. & Ashkenasy, G. Systems Chemistry: Logic Gates, Arithmetic Units, and Network Motifs in Small Networks. *Chem. Eur. J*. **15**, 1765-1775, (2009).

2. Dadon, Z., Wagner, N., Alasibi, S., Samiappan, M., Mukherjee, R. & Ashkenasy, G. Competition and Cooperation in Dynamic Replication Networks. *Chem. Eur. J*. **21**, 648-654, (2015).

3. Wagner, N., Mukherjee, R., Maity, I., Peacock-Lopez, E. & Ashkenasy, G. Bistability and Bifurcation in Minimal Self-replication and Non-enzymatic Catalytic Networks. *ChemPhysChem*. **18**, 1842–1850, (2017).

4. Peacock-Lopez, E., Chemical Oscillations: The Templator Model. *Chem. Educator* **6**, 202-209 (2001).

5. Gurevich, L., Cohen-Luria, R., Wagner, N. & Ashkenasy, G. Robustness of Synthetic Circadian Clocks to Multiple Enviromental Changes. *Chem. Commun.* 51, 5672-5675, (2015).

6. Dadon, Z., Samiappan, M., Shahar, A., Zarivach, R. & Ashkenasy, G. A High-Resolution Structure that Provides Insight into Coiled-Coil Thiodepsipeptide Dynamic Chemistry. *Angew. Chem. Int. Ed.* **52**, 9944-9947, (2013).

7. Mukherjee, R., Cohen-Luria, R., Wagner, N. & Ashkenasy, G. A Bistable Switch in Dynamic Thiodepsipeptide Folding and Template-Directed Ligation. *Angew. Chem. Int. Ed.* **54**, 12452-12456, (2015).
